# Shuhe granule for insomnia: study protocol for a double-blind, randomized, placebo-controlled trial

**DOI:** 10.3389/fphar.2025.1542897

**Published:** 2025-02-24

**Authors:** Yu Gao, Xiangbin Chen, Yuxi Li, Yuchuan Shen, Xinyi Chen, Qing Xu, Mengling Peng, Wen Xu, Jiamin Yuan, Yuanyuan Hu, Xiankun Chen, Biyun Xu, Zhimin Yang

**Affiliations:** ^1^ The Second Clinical Medical College of Guangzhou University of Chinese Medicine, Guangzhou, China; ^2^ The Second Affiliated Hospital of Guangzhou University of Chinese Medicine, Guangdong Provincial Hospital of Chinese Medicine, Guangzhou, China; ^3^ Department of Sleep Disorder, The Third People’s Hospital of Zhongshan, Zhongshan, China; ^4^ State Key Laboratory of Dampness Syndrome of Chinese Medicine, The Second Affiliated Hospital of Guangzhou University of Chinese Medicine, Guangzhou, China; ^5^ Key Unit of Methodology in Clinical Research, Guangdong Provincial Hospital of Chinese Medicine, Guangzhou, Guangdong, China; ^6^ Dep. of Sleep Disorder, The Second affiliated Hospital of Guangzhou University of Chinese Medicine, Guangzhou, China

**Keywords:** Shuhe granule, insomnia, randomized controlled trial, protocol, TCM

## Abstract

**Introduction:**

Modern medical treatment of insomnia is often associated with issues like addiction, drug resistance, and a high risk of relapse post drug withdrawal. To tackle these challenges, the Chinese medicine formula Shuhe granule (SHG) has been employed in insomnia treatment at Guangdong Provincial Hospital of Chinese Medicine. Despite this, there is currently a lack of reliable evidence from evidence-based trials to support the widespread use of SHG in insomnia treatment. Therefore, we have developed a randomized controlled trial to assess the efficacy and safety of SHG in insomnia treatment.

**Method:**

This study will be a single-center, double-blind, randomized, parallel group, placebo-controlled clinical trial involving a total of 160 eligible patients aged between 18 and 65. Participants will be randomly assigned to either the intervention group, receiving SHG, or the placebo group receiving placebo granules. Follow-up visits will occur every 2 weeks from baseline to 2 months. The primary outcome is the perceived insomnia severity measured by the Insomnia Severity Index. The secondary outcome measures include the Pittsburgh Sleep Quality Index, Fatigue Severity Scale, Patient Health Questionnaire, Generalized Anxiety Disorder, Visual Fatigue Analogue Scale, Traditional Chinese medicine syndrome score, and Polysomnography.

**Discussion:**

The results of the study are expected to provide evidence of high methodological and reporting quality on the efficacy and safety of SHG for insomnia.

**Trial Registration Numbers:**

http://itmctr.ccebtcm.org.cn/, ITMCTR2024000035.

## 1 Introduction

Currently, chronic insomnia is emerging as a global epidemic with a significant increase in prevalence in China. Epidemiological surveys have shown that insomnia affects 29.2% of the general population (Shi et al., 2020) and 38.9% of medical staff (Pappa et al., 2020). The consequences of chronic insomnia go beyond sleep disturbances, impacting various aspects of health including physiological, psychological, and cognitive functions. Patients with insomnia experience disrupted sleep patterns leading to reduced sleep quality, impaired daytime functioning, and potential development of mental health issues such as anxiety and depression (Hertenstein et al., 2019; Riemann, 2022; Scott et al., 2021). Moreover, chronic insomnia is linked to compromised immune function, heightened risk of cardiovascular diseases, metabolic imbalances, and increased sensitivity to pain (Yin et al., 2017; Sanjari Moghaddam et al., 2021).

Sleeping pills are commonly used to treat insomnia clinically. While they can provided short-term relief from sleep deprivation, they may also lead to issues like abuse and addiction (Hajak et al., 2003). Chronic insomnia patients using sleeping pills long-term often experience adverse reactions, withdrawal symptoms, and rebound insomnia (Wilt et al., 2016). Moreover, the long-term effects of these medications may be uncertain due to the higher risk of side effects (Wilt et al., 2016). Therefore, apart from traditional treatments, exploring alternative safe and effective treatments for chronic insomnia patients is crucial.

In Traditional Chinese Medicine (TCM), it is believed that good health comes from the balance of Yin and Yang in the body, whereas disease is caused by an excess or deficiency of Yin or Yang, which is referred to as “Yin-Yang disharmony” (Hu et al., 2019). TCM theory posits that sleep is a result of the harmony between Yin and Yang, with Yang entering Yin leading to sleep and Yang exiting Yin leading to wakefulness. It asserts that the root cause of insomnia is often a disharmony between Yin and Yang, particularly an excess of Yang coupled with a deficiency of Yin. In TCM, there is a theory of Zang-Fu organs characterized five Zang organs which comprise solid organs such as the Heart, Spleen, Lung, Kidney, and Liver. Qi and Blood flow between Zang-Fu organs. TCM theory believes that Qi, Blood and Zang-Fu organs are the important material basis of the human body. Therefore, imbalance of qi and blood in the organs is also a key factor causing disease in traditional Chinese medicine. Previous studies have indicated that individuals with chronic insomnia may experience deficiencies in heart and kidney function, as well as imbalances in Qi and Blood due to the prolonged duration of the condition. The pathogenesis has evolved from “Yin-Yang disharmony, Yang exuberance with Yin debilitation” to “deficiency Yang floating upward”. Consequently, the treatment of chronic insomnia necessitates the warming and descending of Yang.

Based on this theory, Shuhe Granules (SHG), a representative Chinese patent medicine, was developed from the extensive clinical expertise of the renowned TCM physician Yang Zhimin. It is an herbal formula comprising of nine medicinal herbs (Table 1). The chemical fingerprint analysis of SHG was performed using ultra-performance liquid chromatography coupled with Q-exactive Orbitrap mass spectrometry, revealing over 100 common chemicals peaks (Supplementary Material 1–5).

**TABLE 1:** The components of the Chinese herbal formula Shuhe.

Herbal name	Dosage (g)	Produced from
*Cinnamomum cassia* (L.) J.Presl [Lauraceae]	15	Dried twig
*Paeonia lactiflora* Pall. [Paeoniaceae]	15	Dried root
*Glycyrrhiza uralensis* Fisch. [Fabaceae]	12	Dried root and rhizome
*Zingiber officinale* Roscoe [Zingiberaceae]	18	Fresh rhizome
*Ziziphus jujuba* Mill. [Rhamnaceae]	18	Dried fruit
*Panax ginseng* C. A. Mey. [Araliaceae]	10	Dried root and rhizome
*Angelica sinensis* (Oliv.) Diels [Apiaceae]	10	Dried root
*Ophiopogon japonicus (Thunb.) Ker Gawl*. [Asparagaceae]	10	Dried root
*Morinda officinalis* How [Rubiaceae]	10	Dried root

Our early single-arm pilot RCT of 62 participants showed that following a 2-week of SHG intervention, the Insomnia Severity Index (ISI) decreased by 15.38%, resulting in an average score reduction of 4.42 points. Additionally, the Pittsburgh Sleep Quality Index (PSQI) decreased by 16.23%, with an average score reduction of 2.63 points. Our findings suggest that ISI may be a more reliable indicator of the effectiveness of SHG compared to PSQI. No adverse events (AEs) were reported. However, there is currently insufficient evidence from evidence-based trials to support the widespread use of SHG for treating insomnia. As a result, we are planning to conduct a well-designed, randomized double-blind controlled clinical trial to further investigate the efficacy and safety of SHG in the treatment of chronic insomnia.

## 2 Methods

### 2.1 Investigational medications

Receive each qualified traditional Chinese medicine piece according to the prescription amount, add it to the extraction tank with drinking water, heat and decoct for extraction twice. For the first time, add drinking water 10 times the amount of the total medicinal materials, heat and extract for 1.5 h. For the second time, add drinking water 8 times the amount of the total medicinal materials, heat and extract for 1 h, combine the secondary filtrate and collect it in a settling tank for later use. This extraction ratio and time has been verified by the best process conditions of Shuxin Anshen Paste (SAP) in the early stage of our team research, and retains the effective ingredients of each herbal medicine to the greatest extent (Ting-yuan, 2024). SHG is modified from SAP, so we retain this extraction ratio. Extract the mixed filtrate in the settling tank to a vacuum concentrator for concentration, decompress it to a relative density of 1.030 ∼ 1.070 (measured at 60°C), collect and put it into a cold storage for later use. In addition, take maltodextrin, add 2–3 times of 80°C ∼ 100°C purified water, stir until completely dissolved, filter with 100 mesh sieve, add it to the qualified extract and mix evenly, heat the prepared mixed dip to 60 ∼ 70°C, spare. Take the above mixed extract, filter it through 100 mesh sieve, place it in a spray dryer and perform a spray drying operation to dry it to a moisture content of 5.0% (quick), collect it and set aside. Take Shuhe granules and spray dry powder and maltodextrin into a three-dimensional motion mixer and mix for 5–10 min to make them uniform. Take the mixed powder and place it in a dry granulator for granulation, pass it through a 14-mesh sieve, and use a 10-mesh/60-mesh vortex vibrating sieve to grind the dry granules. If the powder is qualified, collect it. Last, take the sieved particles and mix at 30 Hz for 3–5 min, collect and set aside.

“Type A extract” are botanical drugs, and their extracts are included in the national or regional pharmacopoeia used as active ingredients in phytopharmaceuticals with regulated medical use (licensed, listed, or registered medicines) (Heinrich et al., 2022). As a Type A extract, SHG is composed of 13 botanical drugs and functions to reconcile Qi and blood, warm and tonify the heart and kidney. Through modern pharmaceutical technology, the decoctions of SHG are then extracted, concentrated, dried, and shaped into granules. The quality control analysis of SHG used ultra-performance liquid chromatography (UPLC) (Heinrich et al., 2020). The daily dosage of SHG is as follows: Neolitsea cassia (L.) Kosterm. [Lauraceae; Guizhi-branch]: 15 g; Paeonia lactiflora Pall. [Paeoniaceae; Baishao-radix]: 15 g; *Zingiber officinale* Roscoe [Zingiberaceae; Shengjiang-rhizome]: 18 g; Glycyrrhiza uralensis Fisch. [Fabaceae; Gancao-radix et rhizome]: 12 g; *Ziziphus jujuba* Mill. [Rhamnaceae; Dazao-fructus]: 18 g; *Panax ginseng* C. A. Mey. [Araliaceae; Renshen-radix et rhizome]: 10 g; *Angelica sinensis* (Oliv.) Diels [Apiaceae; Danggui-radix]: 10 g; *Ophiopogon japonicus (Thunb.) Ker Gawl*. [Asparagaceae; Maidong-radix]: 10 g; *Morinda officinalis* How [Rubiaceae; Bajitian- radix]: 10 g.

### 2.2 Trial design

**FIGURE 1:**
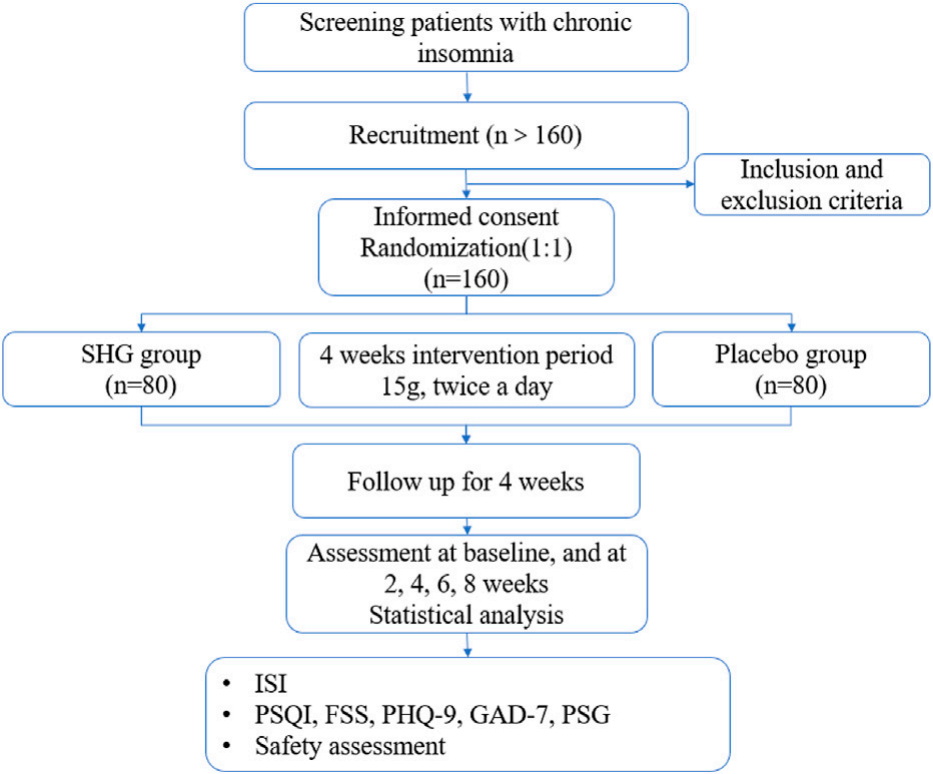
Study flowchart. SHG, Shuhe Granule; ISI, Insomnia Severity Index; PSQI, Pittsburgh Sleep Quality Index; FSS, Fatigue severity scale; PHQ-9, Patient Health Questionnaire; GAD-7, Generalized Anxiety Disorder; PSG, Polysomnography.

This is a single-center, randomized, double-blind, parallel group, placebo-controlled clinical trial. The trial will be conducted in the Guangdong Provincial Hospital of TCM. A total of 160 eligible participants will be randomized into the experimental group (SHG) or the control group (placebo) with a 1: 1 ratio. The duration of the intervention is 4 weeks and the total observation follow-up period will be 8 weeks. Study visits will take place at baseline and at 2, 4, 6, 8 weeks. Each patient will be asked to visit within 3 days of the given time point. All participants will be required to sign the written informed consent (Supplementary Material 6, 7) before randomization. This protocol was compiled in line with the Standard Protocol Items: Recommendations for Interventional Trials (SPIRIT) Guidelines (Supplementary Material 8; Chan et al., 2013). The flowchart of this trial is presented in Figure 1. The schedule of enrollment, interventions, and assessments is as shown in Table 2.

**TABLE 2:** A standard protocol items: recommendation for interventions for trials (SPIRIT).

Study phase time	Baseline	Intervention period		Follow up	
	−1∼0 weeks	2 weeks	4 weeks	6 weeks	8 weeks
Data collection at baseline					
Eligibility screen	√				
Informed consent	√				
Demographic data	√				
Allocation	√				
Medical history	√				
Outcome measurements					
ISI	√	√	√	√	√
PSQI	√		√		√
FSS	√		√		√
GAD-7	√		√		√
PHQ-9	√		√		√
PSG	√		√		
Safety evaluation					
Blood routine	√		√		
Urine routine	√		√		
Liver and kidney function	√		√		
Electrocardiogram	√		√		
AEs		√	√	√	√

ISI, Insomnia Severity Index; PSQI, Pittsburgh Sleep Quality Index; FSS, Fatigue severity scale; PHQ-9, Patient Health Questionnaire; GAD-7, Generalized Anxiety Disorder; PSG, Polysomnography; AEs, adverse events.

“√” Represents the need for implementation at this point in time.

### 2.3 Setting

This study is being conducted in the Treatment and Prevention Center of Guangdong Provincial Hospital of Chinese Medicine. 160 participants will be recruited via putting up posters in the hospital areas. A specialist will be responsible for screening participants and assessing whether they meet with the inclusion criteria. A research assistant will provide the participants with the informed consent form.

### 2.4 Participants

#### 2.4.1 Diagnostic criteria for insomnia

According to the diagnostic criteria for chronic insomnia proposed by American Academy of Sleep Medicine in 2014, participants must meet all items A to F to be included in the study (Sateia, 2014). In accordance with the classification criteria of insomnia proposed by Charles M. Morin in 1993, 8≤ISI≤14, 15≤ISI≤21 and 22≤ISI≤28 represent mild, moderate, and severe insomnia. Only patients with mild to moderate insomnia were included in the study.

#### 2.4.2 Diagnostic criteria for “deficiency of heart and kidney, disorder of qi and blood syndrome” syndrome

Patients have two of the primary symptoms, and more than two of the secondary symptoms, indispensable tongue and pulse listed in the following to be diagnosed with the deficiency of heart and kidney, disorder of qi and blood syndrome (Table 3). Syndrome differentiation will be independently determined by two qualified researchers.

(1) Primary signs and symptoms: insomnia, fatigue.
(2) Secondary signs and symptoms: fear of cold and wind, cold limbs, palpitations, dizziness, spontaneous sweating, scant menstruation, pale lower eyelids or slightly red.
(3) Tongue and pulse: pale red tongue, weak pulse, fine pulse and sunken pulse.

**TABLE 3:** Diagnostic criteria for traditional Chinese medicine syndromes.

Deficiency of heart and kidney, disorder of qi and blood syndrome	
Primary signs and symptoms	1.Insomnia
	2.Fatigue
	The included patients need to have two of the above symptoms
Secondary signs and symptoms	1. Fear of cold and wind
	2. Cold limbs
	3. Palpitations
	4. Dizziness
	5. Spontaneous sweating
	6. Scant menstruation
	7. Pale lower eyelids or slightly red
	The included patients need to have two or more of the above symptoms
Tongue and pulse	1. Pale red tongue
	2. Weak pulse, fine pulse or sunken pulse
	The included patients need to have two of the above symptoms

#### 2.4.3 Inclusion criteria

Participants meeting the following criteria will be included:

(1) The men and women patients are aged 18–65 years.
(2) Patients who meet the diagnostic criteria for insomnia based on the International Classification of Diseases 11th Revision (Harrison et al., 2021).
(3) Patients who meet the diagnostic criteria of insomnia in traditional Chinese medicine.
(4) Patients who meet the deficiency of heart and kidney, disorder of qi and blood syndrome.
(5) Patients who volunteer to participate in the trial and sign the informed consent form.

#### 2.4.4 Exclusion criteria

Participants with any of the following criteria will be excluded:

(1) Meet two of the excluded symptoms
  a. Red lower eyelids.
  b. Feverish hands.
  c. Dry and hard stool.
  d. Thick and slimy fur or dry and coarse fur.
  e. The pulse-to-respiration ratio is greater than 5.3.
(2) Patients who are pregnant, lactating, or plan to become pregnant, and with known mental disorders.
(3) Based on the medical history and consultation, the doctor confirmed that secondary insomnia was caused by other diseases. For example, local pain, restless legs syndrome, sleep apnea syndrome (apnea-hypopnea index, AHI >15/h), acute or chronic heart failure, chronic obstructive pulmonary disease, acute or chronic bronchitis, etc.
(4) Patients with severe depression [Patient Health Questionnaire 9 (PHQ-9) ≥ 15 score].
(5) Patients with severe anxiety [Generalized Anxiety Disorder 7 (GAD-7) ≥ 15 score].
(6) Patients with severe insomnia [Insomnia Severity Index (ISI) ≥ 22 score].
(7) Patients participating in other clinical trials within 4 weeks of the screening period.
(8) Patients with hemoglobin level less than 90 g/L, white blood cell counts less than 3.0 × 10^9^/L, or platelet count less than 100 × 10^9^/L.
(9) Patients with glomerular filtration rate lower than 40 mL/min.
(10) Aspartate aminotransferase (AST) or Alanine aminotransferase (ALT) levels greater than 1.5 times the upper limit of the normal range.
(11) Patients who are allergic to the trial drug.

#### 2.4.5 Withdrawal/termination criteria

Participants will be withdrawn or terminated from the trial if they meet any of the following criteria:

(1). Serious adverse events are reported at any time, and patients are unable to continue.
(2). Patients who present with serious changes or complications related to the disease during the trial.
(3). Patients who cannot comply with the instructions and follow-up procedures of this trial, leading to incomplete medical administration records, or patients who took banned drugs or other medications that may interfere with the efficacy and safety evaluation of this study.
(4). Patients who request to be withdrawn from the trial.

Besides, researchers will record the last time of drug intake and collect evaluation items of participants who discontinue or deviate from intervention protocols.

### 2.5 Randomization and allocation

The random sequence will be generated by the central randomization system from Guangdong Provincial Hospital of Chinese Medicine. Participants will be randomly allocated into either the treatment or placebo group, at a ratio of 1:1. Patients in the treatment group (n = 80) will receive the treatment of SHG, while patients in the placebo group (n = 80) will receive placebo granules, that are identical in appearance to the SHG. Stratified by the central stratification method, we will use SAS 9.2 statistical software to generate random numbers and the random assignment results. The investigators will obtain the randomized number and drug number by a sequential number in the central randomization system. All center numbers, sequence numbers, randomization numbers, and drug numbers will be managed by the statistical unit.

### 2.6 Blinding

This is a double-blind trial in which patients, researchers, outcome assessors, and statistician are all blinded. Blinding codes will be assigned after the randomization operation. This process will be operated by a specially assigned person, and the sequence number of the subject, corresponding random numbers and grouping results will be the primary blinding base. The clinical researcher obtains the subject allocation code through the Internet, and provides corresponding intervention to the subject patient according to the code. Unblinding is permissible in emergency situations when researchers believe that it is necessary to perform any action for patient’s safety. The details of unblinding will be recorded.

### 2.7 Interventions

Both SHG granules and placebo granules will be provided by the Guangzhou Kangyuan Pharmaceutical. The herbal components of Shuhe are shown in Table 1. The chemical fingerprint analysis of SHG were conducted by using an ultra-performance liquid chromatography coupled with Q-exactive Orbitrap mass spectrometry, leading to more than 100 common chemicals peaks. The main ingredients of the placebo are maltodextrin, tartrazine, picrine and lactose. The placebo smell, appearance, taste and weight are consistent with SHG, and will not affect the implementation of blinding. The researcher will administer the drug for 14 days when the participants are enrolled, and again for 14 days during the follow-up visit in the second week. All participants were monitored for 1 day of PSG before and after treatment.

SHG, as a non-decoction granule, should be kept away from direct sunlight to prevent the decomposition or failure of the pharmaceutical ingredients caused by light. Keep the storage environment dry and avoid excessive humidity (relative humidity below 80%) to prevent the granules from absorbing moisture, clumping or mildew. Store in a cool place (generally 10°C–30°C) to avoid changes in drug properties caused by high temperatures. At the same time, SHG will be sealed and stored to prevent the medicine from absorbing moisture in the air or being contaminated by odors, and avoid being stored together with chemical reagents, volatile substances or toxic and harmful substances to avoid adsorbing odors or being contaminated. Regularly check the packaging and appearance of the granules. If any abnormalities such as discoloration, odor, agglomeration, etc., are found, stop use and consult a pharmacist. The sponsor distributes packaging according to random coding, and the usage, batch number, manufacturer and drug number are printed on the outer packaging box.

#### 2.7.1 Intervention group

Patients in the treatment group will receive SHG granules (2 bags at 15 mg each, orally, twice a day, with warm water) for 4 weeks, and the total observation follow-up period will be 8 weeks. In addition, counseling will be provided by research staff about management in insomnia.

#### 2.7.2 Control group

Patients in the control group will receive placebo granules (2 bags at 15 mg each, orally, twice a day, with warm water) for 4 weeks, and the total observation follow-up period will be 8 weeks. In addition, counseling will be provided by research staff about management in insomnia.

If the participants’ condition has not improved during the clinical trial, the life and work are affected, or emotional changes, we will maintain a good communication with the patients, and give the temporary western medicine temporarily with the consent of fully knowledge. We will instruct patients on how to take medication and what precautions to take, and make a registration for taking medication. Observation for 1 week, during the medication, we will still to observe the changes in the condition at any time, and fall off when necessary. If the ISI remains unchanged or increased, but the GAD-7 and PHQ-9 scores increase ≤4 points, Stilnox 10 mg will be administered on an as-needed basis. Conversely, if the GAD-7 or PHQ-9 scores increase by ≥5 points, or if the anxiety and depression factor scores on the Symptom Checklist-90 are severe, Lorazepam 1 mg will be administered on an as-needed basis.

### 2.8 Outcomes and measurements

The time points of the study data collection for outcome measurements are shown in Table 2.

#### 2.8.1 Primary outcome

The primary outcome is the change of ISI score after 4 weeks of intervetion.

ISI consists of seven items, with scores ranging from 0 to 4 for each item, and total scores ranging from 0 to 7 indicating normal sleep, from 8 to 14 indicating mild insomnia, from 15 to 21 indicating moderate insomnia, and from 22 to 28 indicating severe insomnia (Cerri et al., 2023).

#### 2.8.2 Secondary outcomes

Secondary outcomes are the following:

1). Pittsburgh Sleep Quality Index (PSQI). The PSQI is an internationally established tool that is used to evaluate sleep quality. The scale includes seven dimensions that consist of subjective sleep quality, sleep latency, sleep duration, habitual sleep efficiency, sleep disturbances, sleeping medication and daytime dysfunction. The score correlates adversely with sleep quality which a higher score means the sleep quality is worse, and each factor has a score of 0–3 to provide a total score of 21 points (Buysse et al., 1989; Mollayeva et al., 2016).
2). Fatigue severity scale (FSS). FSS is used to measure the degree of fatigue in patients with various diseases and impaction on people’s activities and lifestyles. It has a total of 9 items, each scored on a scale of 1–7, with higher scores being associated with greater fatigue (Kim et al., 2019).
3). Patient Health Questionnaire (PHQ-9). PHQ-9 consists of nine items, with scores ranging from 0 to 3 for each item, and total scores ranging from 0 to 4 points indicating no depression, from 5 to 9 points indicating mild depression, from 10 to 14 points indicating moderate depression, from 15 to 19 indicates moderate to severe depression, and from 20 to 27 indicates severe depression.
4). Generalized Anxiety Disorder (GAD-7). GAD-7 consists of 7 items, each item scores range from 0 to 3 points, the total score ranges from 0 to 4 points indicating no clinical significance of anxiety, from 5 to 9 points indicating mild anxiety, from 10 to 14 points indicating moderate anxiety, and more than 15 points indicating severe anxiety. A total score of 10 or more may indicate anxiety disorder.
5). Polysomnography (PSG). All PSGs are tested using the Australian Kandi Somte-PSG System, including electroencephalogram, electromyogram, eye movements, electrocardiogram, respiratory airflow, snoring, chest and abdominal movements, leg movements, blood oxygen saturation, and pulse rate. Use the above parameters to perform sleep staging, discover and evaluate sleep apnea, and parasomnias (Boulos et al., 2019).

### 2.9 Safety assessments

The interventions will be carried out under the guidance of a researcher. The herbs in the SHG are safe in accordance with the recommended amount in Chinese Pharmacopoeia. We will closely observe the physical conditions and subjective description of the participants. Blood, urine and electrocardiogram analysis as well as liver and kidney function tests will be performed before enrollment and at 4 weeks after enrollment. All adverse events will be recorded on the case report forms (CRFs) and reported to the principal investigator. Any severe adverse events will be reported to the Ethics Committee of Guangdong Hospital of Traditional Chinese Medicine within 24 h. The committee of medical experts will determine whether adverse events are related to the SHG. Guangdong Hospital of Traditional Chinese Medicine will cover the cost of treatment and the corresponding economic compensation for the damage related to the trial. Participants have the right to withdraw their consent to participate in the study at any time for any reason. Other unintended effects of study interventions and study conduct will also be recorded and reported.

### 2.10 Sample size calculation

The sample size is calculated based on the primary endpoint (the changes in ISI). According to the previous exploratory clinical trial (unpublished), the effect size of the treatment group was 24.3%, with a power of 0.8 and alpha of 0.025, a total of 128 patients should be included to detect a difference in ISI score between the SHG and placebo groups. To account for a 20% loss to follow-up, at least 160 patients (80 patients in each group) are planned to be enrolled.

### 2.11 Statistical analysis

To ensure that the baseline characteristics of participants in the SHG treatment and control groups are the same, a propensity score approach will be used. Variables involving factors such as age, gender, smoking and drinking history, and body mass index will be included in a logistic regression model to calculate a propensity score for each participant. Participants using SHG will be matched to control participants based on propensity scores, using greedy matching with a standard caliper width of 0.2 and without case replacement. The two groups’ baseline characteristics will be summarized by descriptive statistics. Baseline demographic variables will be presented as means, medians, or percentages, and using Chi-square test, independent t-test, or Mann-Whitney U test, as appropriate.

The quantitative data will be processed and analyzed by an independent statistician using PASW 18.0 (IBM SPSS Inc., Armonk, New York, United States). We will conduct preliminary analysis on the full analysis population following the Intention-to-Treat (ITT) principle. The full analysis population included all randomized participants who received at least one dose of study drug, but excluded patients who were erroneously included or did not receive any assigned treatment or follow-up. The per-protocol set is a subset of the full analysis set and includes patients who completed the predetermined minimum exposure to trial drug and completed follow-up. Safety analysis will be performed on the randomized participants who received at least one dose of SHG or placebo and were evaluated for safety. Changes from baseline to post-treatment in ISI, PSQI, FSS, PHQ-9 and GAD-7 will be analyzed. The results of the two groups will be analyzed using analysis of covariance with associated 95% confidence intervals. The Bonferroni correction method is used for multiple comparisons. For example, if ISI compares three times, the significant level after correction is 0.05/3 = 0.0167. Only when the P value is less than 0.0167, we think that the difference between the group is statistically significant.

An ITT analysis will be conducted utilizing the imputation method to address missing data. It is anticipated that the proportion of missing data points will be less than 20%. The linear trend at point method will be employed to substitute missing values. In this approach, the existing data series will be regressed against an index variable that is scaled from 1 to n, allowing for the replacement of missing values with their corresponding predicted values. In instances where predicted values cannot be determined, the last observation carried forward (LOCF) method will be implemented. Additionally, a sensitivity analysis will be performed without imputation to evaluate the robustness of the findings.

### 2.12 Data management

Researchers are responsible for the collection of baseline information and trial data. The researchers will ensure that the study schedule is fully explained and participants are aware of the potential risks and benefits of the treatment before consent is obtained. Participants will receive phone calls before each visit to remind them of their time.

### 2.13 Quality control

The researchers will carry out their responsibilities by adhering to the clinical research plan, following standard operating procedures, and confirming all important observations and results to uphold the quality control and quality assurance system of the clinical research. Subject allocation in the study must comply with the random allocation scheme specified in the study design, and the processing grouping code for each subject will be stored by the statistical unit and the researcher in a confidential manner.

All researchers will receive professional training to understand the protocol. The researchers will obey to guidelines of Good Clinical Practice to ensure the safety, blinding and data quality. The researcher should ensure that all information is accurately and lawfully recorded in medical records and CRFs which kept by designated individuals. Two independent investigators will double - checked all CRFs. Data from the CRFs will be inputted into the SPSS by the two data administrator.

The supervisor is responsible for adhering to established operating protocols, facilitating the execution of the research agenda, and ensuring the accuracy and completeness of all data records and reports. Additionally, they are tasked with verifying the proper completion of Case Report Forms (CRFs) and ensuring their alignment with the original data. The supervisor will conduct thorough assessments of the clinical study’s activities and associated documentation to ascertain compliance with the study protocol, standard operating procedures, and pertinent legal requirements.

For the accuracy and authenticity of the data, it is imperative to ensure precise documentation of all laboratory indexes, with an original report copy affixed to the case report form. To assess data accuracy and procedural adherence, medical statisticians will meticulously input research data into the report, maintaining comprehensive and precise records of all data management activities. Adherence to the approved research protocol is paramount, with any deviations duly noted. Any alterations to the research plan must be detailed and submitted to the Ethics Committee for approval prior to implementation.

### 2.14 Ethical considerations

Protocol version: v2.0 was finished on 2 April 2024. This trial has been approved by the Ethics Committee of Guangdong Hospital of Traditional Chinese Medicine (ZF2024-055-01) (Supplementary Material 9) and will be conducted under the supervision of the Ethics Committee. This study was registered on the International Traditional Medicine Clinical Trial Registration Platform on 22 March 2024, with the registration number ITMCTR2024000035. Any modifications to the protocol regarding trial design, eligibility criteria, sample sizes, or significant changes in the study will initially require agreement from the Ethics Committee. Participants will sign the informed consent forms at the baseline and their personal information will remain strictly confidential. Results will be published in a peer-reviewed academic journal. The full protocol, participant-level data, and statistical code are available from the corresponding author upon reasonable request.

### 2.15 Data handling, record keeping, and dissemination

Data entry and management are the responsibilities of the departmental data administrator. The data entered by the administrator adheres to the principles of double entry and mutual verification. Any issues or unforeseen circumstances that arise during data entry must be promptly recorded and reported. Once data entry and verification are complete, the original CRF will be filed in a numbered sequence, and a retrieval directory will be created for reference. Electronic data files will be classified and organized, with multiple backups saved on different disks or recording media to ensure protection from damage. All original documents are retained in accordance with the guidelines outlined in Good Clinical Practice.

Medical records and biological samples could be utilized in subsequent studies of a similar nature. In such instances, ethical approval will be secured prior to the distribution of the materials. Findings from this research will be disseminated through peer-reviewed medical journals, doctoral dissertations, and/or scientific conferences. Nevertheless, any data gathered from this study that could reveal the identity of an individual will be kept confidential and solely employed for this research.

## 3 Discussion

Insomnia is the most common sleep problem in the general population and seriously affects not only people’s physical and mental health but also their quality of life and work efficiency (Patel et al., 2018). Clinical epidemiological studies have suggested that insomnia is associated with hypertension, stroke, depressive disorders, psychotic disorders, dementia, substance abuse disorders and weakened immunity (Sanjari Moghaddam et al., 2021). The etiology of insomnia is complex, and its pathophysiological mechanisms involve genetic, environmental, behavioral and physiological factors. The treatment of insomnia mainly includes Western drug intervention (such as benzodiazepine receptor agonists), cognitive behavioral therapy, and repetitive transcranial stimulation (Wu et al., 2021; Freund and Weber, 2023).

As a complementary and alternative medicine, TCM is widely used in insomnia treatment in China, which has shown the effects of improving clinical symptoms and enhancing the quality of life (Ng and Parakh, 2021). In our previous small sample exploratory clinical study, we found that SHG significantly shorten the time to fall asleep, improved clinical symptoms, and reduced the insomnia severity index. At the same time, no adverse events (AEs) occurred. Therefore, it is necessary to conduct standardized randomized controlled clinical trials to confirm the efficacy and safety of SHG.

TCM is commonly utilized to produce various therapeutic outcomes through the administration of compounded Chinese medicine (Luan et al., 2020). Nevertheless, the standardization of clinical randomized controlled trials involving compound Chinese medicine has encountered numerous obstacles (Tang et al., 2018). One such challenge is the complexity of conducting clinical investigations based on the principles of “syndrome differentiation and treatment” as outlined in TCM theory (Jiang et al., 2012). In this study, we chose syndrome “deficiency of heart and kidney, disorder of qi and blood syndrome” by analyzing TCM syndrome and medication rules of chronic insomnia. In order to maintain the precision of enrollment, the principal investigators and their attending physicians will work together to conduct diagnoses and examinations. Furthermore, strategies such as double blinding and random grouping will be employed to mitigate potential biases. Specifically designated researcher will be responsible for blinding patients’ grouping outcomes and medication allocations. Moreover, an SHG placebo will be manufactured to closely resemble the test drug in appearance, taste, and weight, ensuring that both researchers and patients remain unaware of the drug’s characteristics. These procedures are designed to uphold the integrity and dependability of data collection and result evaluation.

Secondary insomnia is often caused by physical diseases, mental disorders and sleep breathing disorders (McCrae and Lichstein, 2001). At present, TCM is used to treat insomnia mostly for primary insomnia (Ni et al., 2019), so we excluded most of the possible causes of secondary insomnia, such as severe anxiety, severe depression, and secondary factors combined with other diseases.

SHG is modified from the SAP, which has been used by Professor Yang Zhimin to treat patients with chronic insomnia in our center for years. It has been used to coordinate the heart and kidney, and warm and descend of yang. We have examined the efficacy of SAP on rat’s autonomic activity and the efficacy of SAP and sodium pentobarbital on mouse’s sleep and motor coordination. SAP can suppress the rat’s autonomic activity. The synergism of SAP and pentobarbital sodium can promote the mouse’s sleep (Fu-pin, 2015).

In many clinical assessments, ISI is used to measure the severity of insomnia, and PSQI is often used as an index to measure the overall sleep quality (Niu et al., 2023). In this study, the primary outcome is ISI score at week 4. The ISI is a crucial outcome measure in clinical trials assessing the severity of insomnia in patients. The ISI is a validated self-report questionnaire that provides a comprehensive assessment of the impact of insomnia on an individual’s daily functioning, including sleep satisfaction, sleep interference, and daytime impairment (Bastien et al., 2001). ISI focuses on the subjective symptoms of insomnia, the consequences and the degree of distress, and is sensitive to detect changes in sleep conditions brought about by treatment. However, researchers need to be alert to the possibility of false positive results when patients subjectively amplify their insomnia feelings. Therefore, we also conducted PHQ-9 and GAD-7 to assess the degree of anxiety and depression. Current research has found a strong correlation between insomnia and fatigue (Kim et al., 2019). We believe that SHG can improve both patients’ insomnia and fatigue symptoms, so we also conducted evaluations of FSS. At the same time, we conducted PSG examinations before and after the intervention in order to find objective evidence that SHG improved sleep structure.

The study also has certain limitations. The single-center study may not extrapolate the results to other ethnic groups or regions. Sample size and treatment duration are calculated based on our previous exploratory study, which may lead to an overestimation of the efficacy of SHG. Longer follow-up is needed to observe the impact of SHG on clinical prognosis such as the dosage of western medicine and the recurrence rate of insomnia in patients with chronic insomnia.

In conclusion, this trial will provide high-quality evidence for usage of SHG, hoping to reduce the insomnia severity index and improve clinical symptoms and quality of life of patients with insomnia. Whether the results are neutral, negative, or positive, this trial will have clinical implications for patients with chronic insomnia.

## Abbreviations

AEs, Adverse events; CRFs, Case report forms; FSS, Fatigue severity scale; GAD, Generalized Anxiety Disorder; ISI, Insomnia Severity Index; PHQ, Patient Health Questionnaire; PSG, Polysomnography; PSQI, Pittsburgh Sleep Quality Index; SHG, Shuhe Granule; TCM, Traditional Chinese Medicine; SAP, Shuxin Anshen Paste.
