# Supplementary material for: Shuhe granule for insomnia: study protocol for a double-blind, randomized, placebo-controlled trial
Source: Front Pharmacol. 2025 Feb 24;16:1542897. doi: 10.3389/fphar.2025.1542897 (PMC11891157; doi:10.3389/fphar.2025.1542897)
Supplement: Supplementary file 1 [file Supplementaryfile5.pdf]

中药配方颗粒检验报告单

NO:230355

代　　号		品　　名	舒和颗粒（药）
批　　号	2312309	规　　格	每袋装10.6克
依　　据	中国药典2020年版、江阴天江药业有限公司内控质量标准	数　　量	6132袋
收检日期	2024年01月04日	报告日期	2024年01月09日
取样方法	GZ-SOP-ZK-405中药配方颗粒取样操作规程	取样数量	60 袋
生产日期		有效期	2026年11月

检查项目	标　　准	结　　果	单项结论
性　状	本品应为浅黄色至黄棕色颗粒，气微，味微甘微苦微辛。	本品为浅黄色颗粒，气微，味微甘微苦微辛。	符合规定
水　分	≤8.0%	3.3%	符合规定
溶化性	应全部溶化，允许有轻微浑浊。	全部溶化	符合规定
装量差异	装量差异限度±5 %	符合规定	符合规定
粒　度	不能通过一号筛和能通过五号筛的总和不得超过15%	6%	符合规定
微生物限度检查			
需氧菌总数	<10^3 cfu/g	10cfu/g	符合规定
霉菌和酵母菌总数	<10^2 cfu/g	<10cfu/g	符合规定
大肠埃希菌	不得检出/g	未检出	符合规定

（印章：江阴天江药业有限公司 检测报告专用章）

结　　论	本品按中国药典2020年版四部、江阴天江药业有限公司内控质量标准检验，结果符合规定

批准人：[signature]　　审核人：[signature]　　编制人：[signature]
