# Supplementary material for: Shuhe granule for insomnia: study protocol for a double-blind, randomized, placebo-controlled trial
Source: Front Pharmacol. 2025 Feb 24;16:1542897. doi: 10.3389/fphar.2025.1542897 (PMC11891157; doi:10.3389/fphar.2025.1542897)
Supplement: Supplementary file 2 [file Supplementaryfile1.docx]

**1. The composition of Shuhe granule**

| Herbal name | Dosage(g) | Produced from |
| --- | --- | --- |
| *Cinnamomum cassia* (L.) J.Presl [Lauraceae] | 15g | Dried twig |
| *Paeonia lactiflora* Pall. [Paeoniaceae] | 15g | Dried root |
| *Glycyrrhiza uralensis* Fisch. [Fabaceae] | 12g | Dried root and rhizome |
| *Zingiber officinale* Roscoe [Zingiberaceae] | 18g | Fresh rhizome |
| *Ziziphus jujuba* Mill. [Rhamnaceae] | 18g | Dried fruit |
| *Panax ginseng* C. A. Mey. [Araliaceae] | 10g | Dried root and rhizome |
| *Angelica sinensis* (Oliv.) Diels [Apiaceae] | 10g | Dried root |
| *Ophiopogon japonicus* (Thunb.) Ker Gawl. [Asparagaceae] | 10g | Dried root |
| *Morinda officinalis* How [Rubiaceae] | 10g | Dried root |

**2、The preparation of Shuhe granule**

2050 g of raw herb material of Shuhe formula was decocted with 10 times of water for 1.5 h. After removing the decoction, the material was subjected to second decocting with 8 times of water for another 1 hour. The decoction filtrated, combined, and concentrated to the relative density of 1.10 ~ 1.20 (60 °C). The obtained ointment was added 200 g of maltodextrin and fully mixed. After drying, it was crushed into a fine powder, which was further mixed with 20 g of maltodextrin and then made into 1,000 g granules.
