# Supplementary material for: Shuhe granule for insomnia: study protocol for a double-blind, randomized, placebo-controlled trial
Source: Front Pharmacol. 2025 Feb 24;16:1542897. doi: 10.3389/fphar.2025.1542897 (PMC11891157; doi:10.3389/fphar.2025.1542897)
Supplement: Supplementary file 4 [file Supplementaryfile3.docx]

**Quantification of marker compounds of Shuhe particle content**

**Materials and methods**

0.12 g Shuhe granules (batch No. 2207326, 2308301, 240401, 2312309) were weighed and dissolved in 10 mL of 30% methanol. The sample preparation for HPLC is the same as in supplemental file 2. Paeoniflorin (batch No. 110736-202044, China National Institute of Food and Drug Control), glycyrrhizin (batch No. 111610-201908, China National Institute of Food and Drug Control), All of them were dissolved with 30% methanol and prepared into a stock solution with a concentration of 1 mg/mL for each. Paeoniflorin was diluted into 5 µg/mL, 10 µg/mL, 20 µg/mL, 25 µg/mL, 50 µg/mL, 100 µg/mL, 200 µg/mL reference solution successively. Liquiritin stock solution was diluted successively into 2 µg/mL, 5 µg/mL, 10 µg/mL, 20 µg/mL, 40 µg/mL, 60 µg/mL, 80 µg/mL and 100 µg/mL control solution. The HPLC analysis of Shuhe was carried out on an Agilent 1260 HPLC system.

The chromatographic separation was performed using a Luna Su C18 (250×4.60 mm, 5 µm) at 30 ℃, Ultrapure water (A), and acetonitrile (B) was used as the mobile phase for analysis. The flow rate was set at 1.0 mL/min. For paeoniflorin, the elution conditions were carried out by equal degree program with 87% A for 65 min and UV wavelength was 230 nm. For liquiritin, the elution with 85%A for 50min was applied and the target was detected at 280 nm. 10 μL of both sample and reference were injected into the HPLC system for analysis.

The methodology of the HPLC determination of two marker compounds liquiritin and paeoniflorin in this compound formula was evaluated in terms of the specificity, linearity, repeatability and intermediate precision, stability, and accuracy.

**Results**

**1. Methodological evaluation**

1.1 Specificity test

By comparing the chromatograms of reference compounds, Shuhe granule samples and blank sample, two targets liquiritin and paeoniflorin are separated/resolved clearly, indicating that the method has good specificity (figure 1).


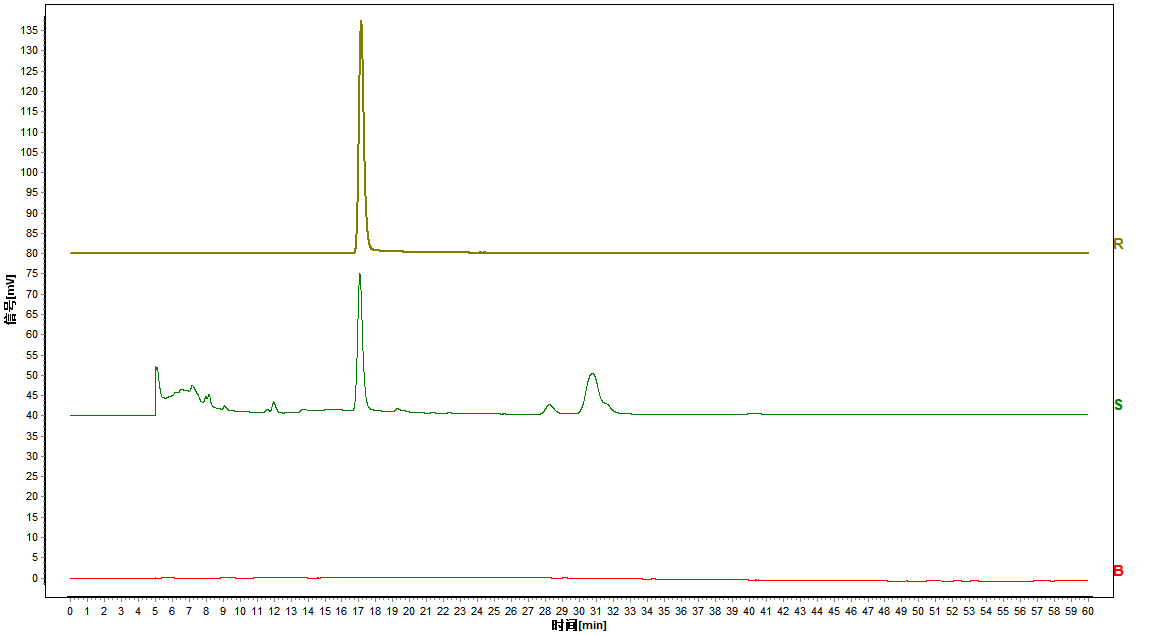


**230nm**

**P**


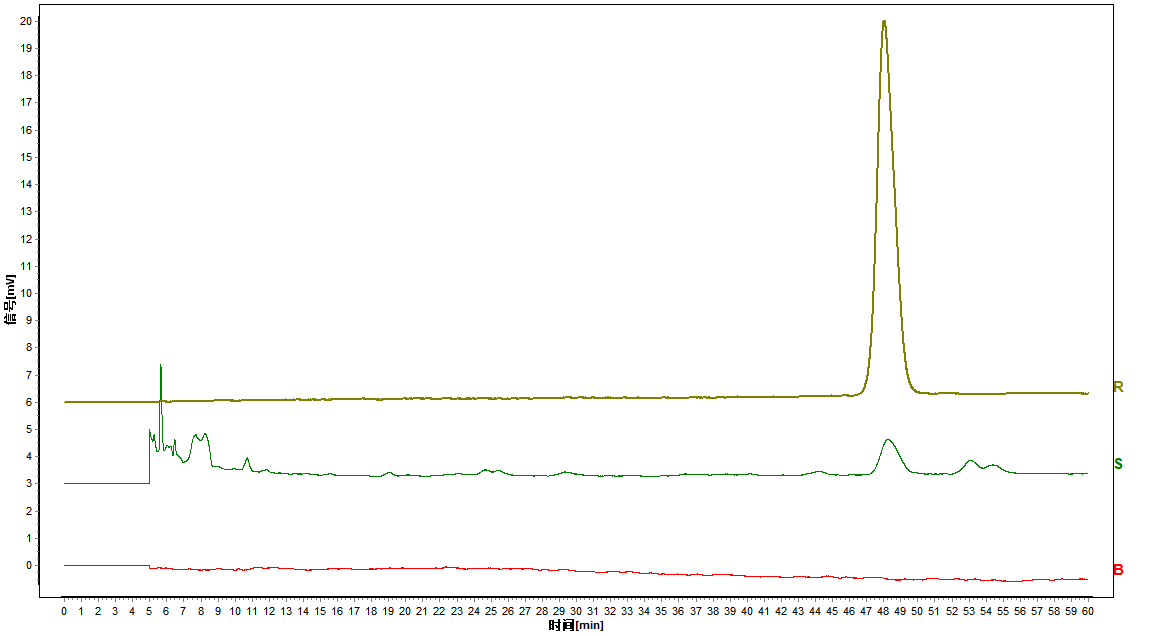


**280nm**

**L**

**Figure 1.** The HPLC chromatograms of reference compounds, Shuhe granule samples and blank sample. (**P:** Paeoniflorin; **L:** liquiritin; **R:** Reference substance; **S:** Shuhe sample; **B:** Blank sample)

1.2 Linearity

The control solution with a series of concentrations was injected according to the above conditions, the injection volume was 10 µL, and linear regression was performed. The regression equation is shown in Table 1, indicating that each component has a good linear relationship within the corresponding concentration range.

**Table 1** The calibration curve (R^2^) and the regression data for the two compounds.

| Analyte | Linear function | R² | Linear Range (µg/mL) |
| --- | --- | --- | --- |
| paeoniflorin | y=9.6548 x-5.0497 | 0.9998 | 4.84~193.60 |
| liquiritin | y=24.2472 x-5.1213 | 1.0000 | 1.90~95.00 |

1.3 Repeatability

For the repeatability testing, The Shuhe sample (2201726) was prepared for six times and tested by one operator at the same condition. The RSD (%) value of each peak area showed there is qualified repeatability (Table 2).

1.4 Intermediate precision.

Intermediate precision was evaluated using the same sample but carried out by two operators with the same equipment. The RSD (%) value of the content of each component was calculated. Results showed there is good precision (Table 2)

1.5 Stability

The stability of the method was carried out within 24h. The Shuhe sample (2201726) was prepared and stored at room temperature. Sample was test at six time points (0, 1, 2, 4, 8, 12, 24 h), respectively. And the RSD (%) of peak areas showed good stability in 24 h (Table 2).

1.6 accuracy

The accuracy was evaluated by using Spike-and-recovery experiment. 0.0600 g of the fine powder of Shuhe sample (2201726) was added with equivalent amounts of reference sample, and then extracted and tested as described in “Materials and methods”. The recovery rate of each analyte were calculated based on the comparison between the tested and theoretical values. And RSD of the recovery rates of six replicates was also recorded (Table 2).

**Table 3**

The repeatability, recoveries and stability data for the two compounds

| Analyte | Intermediate precision RSD (%) | Recovery rate | | Stability  RSD (%) | Repeatability  RSD (%) |
| --- | --- | --- | --- | --- | --- |
|  |  | Mean (%) | RSD (%) |  |  |
| Paeoniflorin | 1.07 | 96.92 | 4.42 | 1.18 | 1.84 |
| liquiritin | 2.14 | 97.53 | 3.43 | 3.98 | 1.47 |

**Sample analysis**

The contents of 2 index active ingredients in 4 batches of Shuhe granules were determined, and the results were calculated and analyzed, as shown in Table 2.

**Table 2**

The contents of two markers in 4 batches of Shuhe granules (mg/g)

| Batch No | Paeoniflorin | liquiritin |
| --- | --- | --- |
| 2201726 | 5.46±0.46 | 1.26±0.18 |
| 2308301 | 5.05±0.46 | 1.05±0.18 |
| 240401 | 5.22±0.46 | 0.90±0.18 |
| 2312309 | 4.39±0.46 | 0.86±0.18 |
| Mean value | 5.03±0.46 | 1.02±0.18 |
| RSD% | 9.05 | 17.96 |
