# Supplementary material for: Shuhe granule for insomnia: study protocol for a double-blind, randomized, placebo-controlled trial
Source: Front Pharmacol. 2025 Feb 24;16:1542897. doi: 10.3389/fphar.2025.1542897 (PMC11891157; doi:10.3389/fphar.2025.1542897)
Supplement: Supplementary file 5 [file Supplementaryfile6.doc]

# 舒和颗粒治疗慢性失眠气血失和、心肾两虚证的单中心、双盲、随机、安慰剂对照临床研究

知情同意书·知情告知页（研究简介）

Information Leaflet for Informed Consent

亲爱的患者：

您的医生已经确诊您患有慢性失眠。

我们将邀请您参加一项舒和颗粒治疗慢性失眠气血失和、心肾两虚证的单中心、双盲、随机、安慰剂对照临床研究。观察舒和颗粒对于慢性失眠人群的干预疗效和安全性。治疗途径是口服药物治疗。

在您决定是否参加这项研究之前，请尽可能仔细阅读以下内容，它可以帮助您了解该项研究以及为何要进行这项研究，研究的程序和期限，参加研究后可能给您带来的益处、风险和不适。如果您愿意，您可以请您的医生给予解释，或者可以和您的家属、朋友一起讨论，帮助您做出决定。

研究介绍

一、研究背景和研究目的

慢性失眠是常见的慢性疾病，一般治疗通过认知行为治疗、镇静催眠药、抗焦虑抑郁药物进行治疗。生活方式控制可能存在依从性不良，口服安眠物可能存在成瘾性或白天功能异常等潜在的副作用。

前期研究表明，舒和颗粒对于慢性失眠患者具有良好的临床疗效，在改善睡眠、提高日间活动度方面具有较好的疗效，且具有良好的停药效果。对照组采用的是舒和颗粒安慰剂，安慰剂主要成分为淀粉、可食用色素等，对照组药物从外观、包装上与治疗组一致，用于对比舒和颗粒的使用效果。

本研究采用单中心、双盲、随机、安慰剂对照试验设计，慢性失眠患者由于病程日久，久病必虚，出现气血失和，心肾两虚的虚损表现，其病机从“阳盛阴衰，阴阳失交”转变为“阳虚虚阳浮越”。因此，慢性失眠应以“温阳潜降”，舒和颗粒以“固精守神，扶阳助阴，调气和血”为法，是由院内制剂舒心安神膏优化而来，已获国家发明专利，由巴戟天、人参、麦冬、芍药、桂枝、当归、生姜、大枣、甘草九味药组成，在临床实践中观察到舒和颗粒对临床慢性失眠病人适用广泛，疗效颇佳，且对日间功能有明显改善作用。

因此，本研究通过实验研究，观察舒和颗粒对慢性失眠的干预效果及安全性，对符合纳入标准的慢性失眠人群进行客观信息采集、临床生化检测、药物干预，分析舒和颗粒的干预效果及使用过程中的安全性。

本项研究已经得到广东省中医院批准。广东省中医院伦理委员会已经审议此项研究是遵从赫尔辛基宣言原则，符合医学伦理的。

二、哪些人适宜参加研究

1.如果您符合以下全部条件可以参加本研究

1. 您年龄在18-65周岁；

2. 您符合西医慢性失眠诊断标准；

3. 您符合中医不寐诊断标准；

4. 您符合气血失和、心肾两虚证；

5. 签署知情同意书自愿参与此项课题研究。

2. 但如果您同时存在以下任意一种情况则不宜参加本研究，因为合并这些情况参加研究不仅浪费您的时间，也会影响研究结果的科学性：

（1）符合排除症状任意一条；

（2）您准备怀孕或孕期或哺乳期或需要陪伴小孩睡觉的人群；或合并其他精神疾病者；

（3）根据您的病史与问诊，医生确认由其它疾病引起的继发性失眠。例如：局部的疼痛、不安腿综合征、睡眠呼吸暂停综合征（呼吸暂停-低通气指数apnea-hypopnea index，AHI＞15/h）、急慢性心力衰竭、慢性阻塞性肺病、急慢性支气管炎；

（4）根据抑郁自评工具（PHQ-9）诊断您为重度抑郁患者（总分≥15）；

（5）根据广泛性焦虑量表（GAD-7）诊断您为重度焦虑患者（总分≥15）；

（6）根据失眠严重程度指数 (ISI) 判定为重度失眠的患者（总分≥22）；

（7）您在过去30天内参加过其它药物临床试验者；

（8）您的血红蛋白水平不到90 g/L，白细胞计数小于3.0×10^9/L，或血小板计数低于100×10^9/L；

（9）您的肾小球滤过率低于40ml/min；

（10）您的天冬氨酸氨基转移酶或丙氨酸氨基转移酶高于正常范围上限1.5倍。

您的研究医生会对您进行评估，并告诉您是否适宜参加本研究。

三、如果参加研究将需要做什么

1、在您入选研究前，您将接受以下检查以确定您是否可以参加研究：

医生将询问、记录您的病史，对您进行睡眠质量问卷等调查。您需要进行血常规，肝功能，肾功能等理化检查。

2、若您以上检查合格，将按以下步骤进行研究

研究开始前您需要停用您既往和睡眠有关的药物及疗法7天。

研究开始时，您需要来医院将进行睡眠质量问卷调查，心电图、尿常规等理化检查，并进行多导睡眠监测。若您符合条件，将根据计算机提供的随机数字，决定您接受舒和颗粒或安慰剂治疗，参与这项研究的患者均有50%的可能性被分入这两个不同的组，您和您的医生都无法事先知道而选择任何一种治疗方法。治疗和随访将持续8周，舒和颗粒治疗组服用舒和颗粒，对照组服用舒和颗粒安慰剂，每天服用2次，每次热水150ml冲服，餐后1小时口服。研究期间禁用西药精神类药物、中药汤剂及其他剂型、如心理治疗、重复经颅磁刺激、经颅直流电刺激、针灸疗法、生物反馈治疗、音乐疗法、认知行为疗法。

研究第4周±3天：治疗结束了，您应该到医院就诊，医生将询问记录您病情的变化，给您进行睡眠质量问卷调查，心电图、血常规，尿常规，肝功能，肾功能等理化检查，及多导睡眠监测。

研究第6周±3天、8周±3天，您需配合随访工作者进行随访。

如果在研究期间，若患者在参加临床试验期间出现病情未见好转且患者感到痛苦、生活工作受到影响。在和您沟通后，仍不能缓解您的问题，会在充分知情同意下，可临时性给予一些西药。

本研究将在0周、4周±3天留取您的血液、尿液、粪便标本，整个研究过程共留取2次。均按标准操作规程进行保存，保存时长为本研究结束后10年，到达保存年限后将对标本进行销毁，您有权要求取回组织样本。其中血液、尿液包括检查性和非研究性，粪便为研究性。

3.需要您配合的其他事项

您需要按医生和您的约定的时间来医院就诊。您的随访非常重要，因为医生将判断您接受的治疗是否真正起作用。

您需要按医生指导用药，您在每次随访时都必须归还未用完的药物及其包装，并将正在服用的其他药物带来，包括您有其他合并疾病须继续服用的药物。

在研究期间您不能和合并其他中西医治疗和干预，或保持原来的治疗和干预不变。如您需要增加其他治疗，请事先与您的医生取得联系。

关于饮食、生活起居的规定：按照既往饮食生活起居进行。

4.您参加试验可能被终止的预期情况和/或原因

4.1研究者从医学角度考虑受试者有必要中止试验

4.2 您自己要求停止试验

4.3您对方案依从性不佳，小于80%。

四、参加研究可能的受益

您和社会将可能从本项研究中受益。此种受益包括您的生存质量有可能获得改善，以及本项研究可能明确舒和颗粒对于慢性失眠的疗效和安全性，以用于患有相似病情的其他病人。

五、参加研究可能的不良反应、风险和不适、不方便

所有治疗方法都有可能产生副作用。研究采用的舒和颗粒在服用期间，可能出现口腔溃疡、咽痛、口苦等情况，一般可自行消失。

如果在研究中您出现任何不适，或病情发生新的变化，或任何意外情况，不管是否与治疗方法有关，均应及时通知您的医生，他将对此作出判断和医疗处理。

医生将尽全力预防和治疗由于本研究可能带来的伤害。

您在研究期间需要按时到医院随访，做一些理化检查，这些都有可能给您造成麻烦或带来不便。

血样：采集血样时可能略有不适。抽血可能引起的副作用包括昏眩、静脉炎症、疼痛、 瘀伤，或者穿刺部位出血。也有很小的感染的可能性。本研究8周时间内一共需要做2次血常规和肝肾功能及尿液常规检查，共需要采集约40ml左右血样，约6茶匙，留取2管大便，留取小便2管。

此外，任何治疗都有可能出现无效情况，以及因治疗无效或者因合并其他疾病等原因而导致病情继续发展。这是每个就医患者都将面临的治疗风险，即使不参加本项临床研究，治疗风险都将存在。在研究期间，如果医生发现本项研究所采取的治疗措施无效，将会终止研究，改用其他可能有效的治疗措施。

六、有关费用

课题组将支付您参加本项研究期间所做的与研究有关的检查（血常规、尿常规、肝功能、肾功能、心电图、多导睡眠）费用，入组筛选的检查费用（血常规、肝功能、肾功能）也同时由课题组支付，并免费提供研究用药。此外，如果按照研究方案完成研究流程，您将获得交通补助，每次50元，共300元，交通补助在研究结束时按研究完成度发放。

如果发生与试验相关的损害，课题组将支付您的医疗费用。如果严重不良反应住院医疗，课题组还将按照法律法规规定给予相应的经济补偿。

如果您同时合并其他疾病所需的治疗和检查，如血脂血糖检测将不在免费的范围之内。

七、个人信息保密的吗？

您的医疗记录（研究病历/CRF、化验单等）将完整地保存在医院，医生会将化验检查结果记录在您的门诊病历上。研究者、申办者代表（如适用）、伦理委员会、科研管理部门或政府管理部门等将被允许查阅您的医疗记录。任何有关本项研究结果的公开报告将不会披露您的个人身份。我们将在法律允许的范围内，尽一切努力保护您个人医疗资料的隐私。

关于生物样本的收集，分别于治疗的第0天、4周±3天或退出试验时点收集患者5-10ml的血清、全血、PBMC标本，10ml尿液样本，2管粪便标本，2管舌苔标本。均按标准操作流程进行保存于广东省中医药生物样本库，保存时长为本研究结束后10年，到达保存年限后将对标本进行销毁。受试者有权要求取回组织样本。您提供的样本将被编码，即采用代码号识别样本而非您的名字或者其他个人信息。只有个别研究者及授权的其他人员才能够从该代码识别您的姓名。未来进行研究的其他研究者或您个人将不能得知您样本用于哪些研究，也不能知道哪些数据是利用您的样本产生的。您有权拒绝剩余样本被保存，拒绝签署本知情同意不会影响您的任何权益，也不会影响您的正常治疗。今后您可以随时通过联系您的研究者，撤回同意，要求立即销毁您的生物样本。但请您注意，如生物样本已经采用了匿名化处理，无法追溯到个人信息，则可能无法撤回。原则上生物样本和相关数据将保存于本单位或相关科研机构。如果您的数据根据研究需要须转移给合作单位或第三方机构，我们会对合作单位或第三方的资质进行审核，并在合作协议中规定相关数据仅可用于科学研究，不得擅自用于商业用途，且数据的使用须严格按照知情同意、研究方案和相关法律法规的规定。

八、怎样获得更多的信息？

您可以在任何时间提出有关本项研究的任何问题。您的医生或研究者将给您留下他/她的电话号码以便能回答您的问题。

如果您对参加研究有任何抱怨，请联系广东省中医院伦理委员会办公室（联系电话：020-81887233-35943）。

如果在研究过程中有任何重要的新信息，可能影响您继续参加研究的意愿时，您的医生会及时通知您。

九、可以自愿选择参加研究和中途退出研究

是否参加研究完全取决于您的自愿。您可以拒绝参加此项研究，或在研究过程中的任何时间退出本研究，这都不会影响您和医生间的关系，都不会影响对您的医疗有其他方面利益的损失。

您的医生或研究者出于对您的最大利益考虑，可能会随时终止您参加本项研究。

如果您不参加本项研究，或中途退出研究，还有很多其他可替代的治疗方法，如认知行为疗法。您不必为了治疗您的疾病而选择参加本项研究。

如果您因为任何原因从研究中退出，您可能被询问有关您参加研究的情况。如果医生认为需要，您可能被要求进行实验室检查和体格检查。这对保护您的健康十分有利。

十、现在该做什么？

在您做出参加研究的决定前，请尽可能向您的医生询问有关问题，直至您对本项研究完全理解。

是否参加本项研究由您自己决定。您可以和您的家人或者朋友讨论后再做出决定。

感谢您阅读以上材料。如果您决定参加本项研究，请告诉您的医生或研究助理，他她会为您安排一切有关研究的事务。

请您保留这份资料。

知情同意书·同意签字页

Signature Leaflet for Informed Consent

**项目名称**：舒和颗粒治疗慢性失眠气血失和、心肾两虚证的单中心、双盲、随机、安慰剂对照临床研究

**申办者/课题下达单位**：广东省中医院

**伦理审查批件号**：ZF2024-055-01

同意声明

我已经阅读了上述有关本研究的介绍，而且有机会就此项研究与医生讨论并提出问题。我提出的所有问题都得到了满意的答复。

我知道参加本研究可能产生的风险和受益。我知晓参加研究是自愿的，我确认已有充足时间对此进行考虑，而且明白：

● 我随时可以向医生咨询更多的信息。

● 我可以随时退出本研究，而且不会受到歧视或报复，医疗待遇与权益不会受到影响。

我同样清楚，如果我中途退出本研究，特别是由于药物的原因使我退出研究时，我若将病情变化告诉医生，完成相应的体格检查和理化检查，这将对我本人和整个研究十分有利。

如果因患病我需要采取任何其他的药物治疗，我会在事先征求医生的意见，或在事后如实告诉医生。

我同意药品监督管理部门、伦理委员会或申办者代表查阅我的研究资料。

我将获得一份经过签名并注明日期的知情同意书副本。

最后，我决定同意参加本项研究。

受试者 签名： 日期： 联系电话：

监护人/授权委托人 签名： 与受试者关系： 日期：

（注：受试者因无行为能力等原因不能签署知情同意的，由其监护人或授权委托人签署）

我同意□ 或拒绝□ 除本研究以外的其他研究利用我的医疗记录和生物标本。

受试者 签名： 日期：

监护人/授权委托人 签名： 与受试者关系： 日期：

（注：受试者因无行为能力等原因不能签署知情同意的，由其监护人或授权委托人签署）

我确认已向受试者解释了本试验的详细情况，包括其权利以及可能的受益和风险，并给其一份签署过的知情同意书副本。

研究者 签名： 日期：

研究者的工作电话： 手机号：

伦理委员会办公室联系电话：020-81887233-35943
