# Supplementary material for: Shuhe granule for insomnia: study protocol for a double-blind, randomized, placebo-controlled trial
Source: Front Pharmacol. 2025 Feb 24;16:1542897. doi: 10.3389/fphar.2025.1542897 (PMC11891157; doi:10.3389/fphar.2025.1542897)
Supplement: Supplementary file 6 [file Supplementaryfile7.doc]

**Shuhe granule for insomnia: Study protocol for a double-blind,** **randomized, placebo-controlled trial**

**Information Leaflet for Informed Consent**

**Dear patient,**

Your doctor has confirmed that you are suffering from insomnia.

We will invite you to participate in a single-center, double-blind, randomized, placebo-controlled clinical study of Shuhe Granules in the treatment of chronic insomnia with Qi-blood disharmony and heart-kidney deficiency. Observe the intervention efficacy and safety of Shuhe Granules for people with chronic insomnia. The treatment route is oral medication.

Before you decide whether to participate in this study, please read the following content as carefully as possible. It may help you understand the study and why it is being conducted, the procedures and duration of the study, and the possible benefits, risks and discomfort that may be brought to you after participating in the study. If you want, you can ask your doctor for an explanation, or you can discuss it with your family and friends to help you make a decision.

**Study introduction**

Ⅰ. Research background and research purpose.

Chronic insomnia is a common chronic disease, and is generally treated with cognitive behavioral therapy, sedative-hypnotics, and anti-anxiety and depression drugs. Lifestyle controls may have poor compliance, and oral sleeping pills may have potential side effects such as addiction or daytime dysfunction.

Preliminary studies have shown that Shuhe Granules have good clinical efficacy for patients with chronic insomnia in improving sleep and increasing daytime activity, and have good drug withdrawal effects. The control group uses Shuhe Granules placebo. The main ingredients of the placebo are starch, edible pigments, etc. The appearance and packaging of the drugs in the control group are consistent with those of the treatment group, which is used to compare the effects of Shuhe Granules.

This study adopts a single-center, double-blind, randomized, placebo-controlled trial design. According to previous research, patients with chronic insomnia will suffer from deficiency of heart and kidney, and disharmony of qi and blood due to the prolonged course of the disease. The pathogenesis has changed from " yin-yang disharmony, yang exuberance with yin debilitation " to " deficiency yang floating upward ". Therefore, the treatment of chronic insomnia also requires warming and descending of Yang. Shuhe granules are based on the method of "consolidating the spirit and guarding the mind, supporting yang and yin, regulating qi and blood". It is optimized from the hospital preparation Shuxin Anshen Ointment, which has been awarded the national invention Patented, it consists of nine medicinal herbs: *Morinda officinalis* How, *Panax ginseng* C. A. Meyer, *Ophiopogon japonicus* (L. f) Ker-Gawl, *Paeonia lactiflora* Pall, *Cinnamomum cassia* Presl, *Angelica sinensis* (Oliv.) Diels, *Zingiber officinale* Rosc, *Ziziphus jujuba* Mill, and *Glycyrrhiza uralensis* Fisch. In clinical practice, it has been observed that Shuhe granules are widely applicable to clinical patients with chronic insomnia and have good curative effects, and has a significant improvement effect on daytime function.

Therefore, this study uses experimental research to observe the intervention effect and safety of Shuhe Granules on chronic insomnia, conducts objective information collection, clinical biochemical testing, and drug intervention on chronic insomnia people who meet the inclusion criteria, and analyzes the intervention effect and safety of Shuhe Granules.

This study has been approved by the Guangdong Provincial Hospital of Traditional Chinese Medicine. The Ethics Committee of Guangdong Provincial Hospital of Traditional Chinese Medicine has reviewed that this study complies with the principles of the Declaration of Helsinki and is in line with medical ethics.

Ⅱ. Who should not participate in the study?

1. You can participate in this study if you meet all of the following conditions:

1). The men and women patients are aged 18–65 years.

2). Patients who meet the diagnostic criteria for insomnia based on the International Classification of Diseases 11th Revision.

3). Patients who meet the diagnostic criteria of insomnia in traditional Chinese medicine.

4). Patients who meet the deficiency of heart and kidney, disorder of qi and blood syndrome.

5). Patients who volunteer to participate in the trial and sign the informed consent form.

2. However, you should not participate in this study if you have any of the following conditions at the same time, because combining these conditions to participate in the research will not only waste your time, but also affect the scientific nature of the research results:

1). Meet any of the excluded symptoms

2). Patients who are pregnant, lactating, or plan to become pregnant, and with known mental disorders.

3). Based on the medical history and consultation, the doctor confirmed that secondary insomnia was caused by other diseases. For example: local pain, restless legs syndrome, sleep apnea syndrome (apnea-hypopnea index, AHI > 15/h), acute or chronic heart failure, chronic obstructive pulmonary disease, acute or chronic bronchitis, etc.

4). Patients with severe depression [Patient Health Questionnaire 9 (PHQ-9) ≥ 15 score].

5). Patients with severe anxiety [Generalized Anxiety Disorder 7 (GAD-7) ≥ 15 score].

6). Patients with severe insomnia [Insomnia Severe Index (ISI) ≥ 22 score].

7). Patients participating in other clinical trials within 4 weeks of the screening period.

8). Patients with hemoglobin level less than 90g/L, white blood cell count less than 3.0×10^9^/L, or platelet count less than 100×109/L.

9). Patients with glomerular filtration rate lower than 40ml/min.

10). Aspartate aminotransferase (AST) or Alanine aminotransferase (ALT) levels greater than 1.5 times the upper limit of the normal range.

Your study doctor will evaluate you and tell you whether it is appropriate for you to participate in this study.

Ⅲ. What will you need to do if you participate in the study?

1. Before you are selected for the study, you will take the following examination to determine whether you can attend the study:

The doctor will ask and record your medical history, and conduct surveys such as sleep quality questionnaires on you. You need to undergo physical and chemical tests such as blood routine, liver function, and kidney function.

2. If you are enrolled in the study, the following steps will be conducted:

You will need to stop taking any sleep-related medications and treatments for 7 days before the study begins.

At the beginning of the study, you need to come to the hospital for a sleep quality questionnaire, electrocardiogram, routine urine and other physical and chemical examinations, and polysomnography. If you are eligible, you will be decided to receive Shuhe Granules or placebo treatment based on the random numbers provided by the computer. Patients participating in this study have a 50% probability of being divided into these two different groups. You and your No doctor can choose any treatment method without knowing it in advance. The treatment and follow-up will last for 8 weeks. The Shuhe Granules treatment group will take Shuhe Granules, while the control group will take Shuhe Granules placebo, twice a day, with 150ml of hot water each time, and orally administered 1 hour after meals. During the study period, Western psychotropic drugs, traditional Chinese medicine decoctions and other dosage forms, such as psychotherapy, repetitive transcranial magnetic stimulation, transcranial direct current stimulation, acupuncture therapy, biofeedback therapy, music therapy, and cognitive behavioral therapy, are prohibited.

Study week 4 ± 3 days: After treatment, you should go to the hospital for treatment. The doctor will ask and record the changes in your condition, and give you a sleep quality questionnaire, electrocardiogram, blood routine, urine routine, liver function, kidney function and other physical and chemical examination, and polysomnography.

At the 6th week ± 3 days and the 8th week ± 3 days of the study, you need to cooperate with the follow-up staff for follow-up.

During the research period, if your condition does not improve while participating in the clinical trial and the patient feels pain, life and work are affected, and your problem still cannot be alleviated after communication, some Western medicines can be temporarily given with full informed consent.

In this study, your blood, urine, and feces samples will be collected at 0 weeks and 4 weeks ± 3 days. A total of 2 samples will be collected during the entire study process. They are all stored in accordance with standard operating procedures, and the storage period is 10 years after the end of this study. After the storage period is reached, the specimens will be destroyed, and you have the right to request the retrieval of tissue samples. Among them, blood and urine include examination and non-research, and feces is research.

3. Other matters requiring your cooperation.

You need to come to the hospital for treatment at the time agreed upon by the doctor and you. Your follow-up visits are very important because your doctor will determine whether the treatment you are receiving is actually working.

You need to take medicines as directed by your doctor. You must return unused medicines and their packaging at each follow-up visit, and bring other medicines you are taking, including medicines you must continue to take if you have other comorbidities.

During the study period, you cannot combine other Chinese and Western medicine treatments and interventions, or keep the original treatments and interventions unchanged. If you need to add other treatments, please contact your doctor in advance.

Regulations on diet and daily life: Follow the previous diet and daily life.

4. Expected circumstances and/or reasons why your participation in the trial may be terminated:

4.1 The researcher considers it necessary for the subject to terminate the trial from a medical perspective.

4.2 You request to stop the trial.

4.3 Your compliance with the protocol is poor, less than 80%.

Ⅳ. Potential benefits from participating in the study.

You and society may benefit from this research. Such benefits include the possibility that your quality of life will be improved, and this study may clarify the efficacy and safety of Shuhe Granules for chronic insomnia so that it can be used for other patients with similar conditions.

Ⅴ. Potential adverse reactions, risks, discomfort, and inconvenience to participate in the study.

All treatments have the potential to cause side effects. During the period of taking the Shuhe granules used in the study, oral ulcers, sore throat, and bitter mouth may occur, which generally disappear on their own.

If you experience any discomfort during the study, or new changes in your condition, or any unexpected circumstances, whether related to the treatment method or not, you should notify your doctor in time, and he will make judgment and medical treatment.

Physicians will make every effort to prevent and treat harm that may result from this study.

During the study period, you need to go to the hospital for follow-up visits and do some physical and chemical examinations on time, which may cause trouble or inconvenience to you.

Blood sample: There may be some discomfort when taking a blood sample. Possible side effects of having your blood drawn include dizziness, inflammation of the veins, pain, bruising, or bleeding at the puncture site. There is also a small chance of infection. This study requires a total of 2 blood routine tests, liver and kidney function and urine routine tests within 8 weeks. A total of about 40ml of blood samples, about 6 teaspoons, need to be collected, 2 tubes of stool and 2 tubes of urine need to be collected.

In addition, any treatment may be ineffective, and the disease may continue to progress due to ineffective treatment or comorbidity with other diseases. This is a treatment risk that every medical patient will face, even if they do not participate in this clinical study, treatment risks will exist. During the study, if the doctor finds that the treatment measures taken in this study are ineffective, the study will be terminated and other treatments that may be effective will be used.

Ⅵ. Related expenses.

The research team will pay for the research-related examinations (blood routine, urine routine, liver function, renal function, electrocardiogram, polysomnography) performed during your participation in this study, as well as the costs for enrollment screening examinations (blood routine, liver function, renal function) are also paid by the research team, and study drugs are provided free of charge. In addition, if you complete the research process according to the research plan, you will receive a transportation subsidy of 50 yuan each time, totaling 300 yuan. The transportation subsidy will be paid at the end of the research based on the completion of the research.

If trial-related damage occurs, the research team will pay your medical expenses. If serious adverse reactions result in hospitalization, the research team will also provide corresponding financial compensation in accordance with laws and regulations.

If you also require treatment and examinations for other diseases, such as blood lipid and blood sugar testing, they will not be included in the free scope.

Ⅶ. Is the personal information kept confidential?

Your medical records (research records/CRF, laboratory test orders, etc.) will be kept completely at the hospital, and the doctor will record the laboratory examination results in your outpatient medical record. Researchers, sponsor representatives (if applicable), ethics committees, scientific research management departments or government management departments will be allowed to access your medical records. Any public reporting of the results of this study will not disclose your personal identity. We will make every effort to protect the privacy of your personal medical information to the extent permitted by law.

Regarding the collection of biological samples, 5-10 ml of serum, whole blood, PBMC specimens, 10 ml of urine samples, 2 tubes of stool specimens, and 2 tubes of stool specimens were collected from patients on day 0, 4 weeks ± 3 days of treatment, or at the time of withdrawal from the trial. Tongue coating specimen. All samples will be stored in the Guangdong Provincial Traditional Chinese Medicine Biosample Bank in accordance with standard operating procedures. The storage period will be 10 years after the end of this study. The specimens will be destroyed after reaching the storage period. Subjects have the right to request retrieval of tissue samples. The samples you provide will be coded, using a code number to identify the sample rather than your name or other personal information. Only individual researchers and authorized others will be able to identify your name from this code. Other researchers conducting future studies, or you personally, will not be able to know which studies your samples were used for, or what data were generated using your samples. You have the right to refuse the remaining samples to be stored. Refusal to sign this informed consent will not affect any of your rights or your normal treatment. You may withdraw your consent and request the immediate destruction of your biological samples at any time in the future by contacting your investigator. However, please note that if the biological sample has been anonymized and cannot be traced back to personal information, it may not be possible to withdraw it. In principle, biological samples and related data will be stored in the unit or relevant scientific research institutions. If your data needs to be transferred to a cooperative unit or a third party for research needs, we will review the qualifications of the cooperative unit or third party and stipulate in the cooperation agreement that the relevant data can only be used for scientific research and shall not be used for commercial purposes without authorization, and the use of data must strictly comply with the provisions of informed consent, research protocols and relevant laws and regulations.

Ⅷ. How do you get more information?

You can ask any questions about this study at any time. Your doctor or researcher will leave you his/her phone number so he/she can answer your questions.

If you have any complaints about participating in the study, please contact the Ethics Committee Office of Guangdong Provincial Hospital of Traditional Chinese Medicine (telephone number: 020-81887233-35943).

Your doctor will notify you promptly if any important new information becomes available during the study that may affect your willingness to continue participating in the study.

Ⅸ. You can voluntarily choose to participate in the study and withdraw from the study midway.

Participation in research is entirely voluntary. You can refuse to participate in this study, or withdraw from this study at any time during the study. This will not affect the relationship between you and the doctor, nor will it affect the loss of other benefits to your medical care.

Your doctor or researcher may terminate your participation in this study at any time out of consideration for your best interests. If you do not take part in this study, or drop out, there are many alternative treatments available, such as cognitive behavioral therapy. You do not have to choose to participate in this study in order to treat your disease.

If you withdraw from the study for any reason, you may be asked about your participation in the study. You may be asked to undergo laboratory tests and a physical examination if your doctor deems it necessary. This is great for protecting your health.

Ⅹ. What should you do now?

Before you make a decision to participate in a study, ask your doctor as many questions as possible until you fully understand the study.

It is your decision whether to participate in this study. You can make a decision after discussing it with your family or friends.

Thank you for reading the above material. If you decide to participate in this study, please tell your doctor or research assistant and he or she will make all study-related arrangements for you.

Please keep this information.

Signature Leaflet for Informed Consent

**Name of clinical research project:** A single-center, double-blind, randomized, placebo-controlled clinical study of Shuhe Granules in the treatment of chronic insomnia with Qi-blood disharmony and heart-kidney deficiency.

Applicant: Guangdong Provincial Hospital of Traditional Chinese Medicine.

Ethics Review Approval No.: ZF2024-055-01

**Consent to the declaration**

I have read the above introduction on this study and have the opportunity to discuss and raise questions with physicians on this study. All the questions I have raised have been satisfactorily answered.

I know the possible risks and benefits of participation in this study. I know that participation in the study is voluntary and I confirm that there is ample time to consider this and understand:

● I can consult my doctor for more information at any time.

● I can withdraw from this study at any time without discrimination or retaliation, and medical treatment and interests will not be affected.

I am equally clear that if I quit the study, especially due to the drug if I told the doctor the changes and completed the corresponding physical and physical examination, it would be very beneficial for me and the whole study.

If I need any other medication due to a changing condition, I will ask the doctor in advance or tell the doctor truthfully after that.

I consent to access to my research data by the drug regulatory authorities, ethics committee, or sponsor representatives.

I will obtain a signed and dated copy of the informed consent form.

Finally, I decided to agree to participate in this study.

Patient signature: Date: Patient contact number:

Signature of guardian/authorized person: Relationship:

(Note: If the subject is unable to sign the informed consent due to incapacity or other reasons, his/her guardian or authorized person shall sign it)

Agree□ Reject□ Research other than this study utilizes my medical records and biological specimens.

Patient signature： Date：

Signature of guardian/authorized person:  Relationship: Date:

(Note: If the subject is unable to sign the informed consent due to incapacity or other reasons, his/her guardian or authorized person shall sign it)

I confirm the details of the trial, including its rights and possible benefits and risks, and give patients a signed copy of the signed informed consent form.

Researcher signature： Date:

Researcher contact number： Phone number：

Ethics committee office contact number: 020-81887233-35943
