# Supplementary material for: Shuhe granule for insomnia: study protocol for a double-blind, randomized, placebo-controlled trial
Source: Front Pharmacol. 2025 Feb 24;16:1542897. doi: 10.3389/fphar.2025.1542897 (PMC11891157; doi:10.3389/fphar.2025.1542897)
Supplement: Supplementary file 7 [file Supplementaryfile9.pdf]

广东省中医院伦理委员会

Ethics Committee of Guangdong Provincial Hospital of Chinese Medicine

伦理审查批件

Approval Notice

批件号：广东省中医院伦理委员会ZF2024-055-01

审查日期	初审：2024年03月01日；		
审查地点	广东省中医院研修楼20楼2005-2006（长形会议室）		
临床研究批文	无		
临床研究项目	舒和颗粒治疗慢性失眠气血失和、心肾两虚证的单中心、双盲、随机、安慰剂对照临床研究		
审查文件	1. 初始审查申请表包括研究经济利益声明<br>2. 研究方案(版本号/日期：1.0/20240206)<br>3. 向受试者提供的知情同意书（包括研究简介和签字页）(版本号/日期：1.0/20240206)<br>4. 临床病例报告表(版本号/日期：1.0/20240206)<br>5. 主要研究者简历<br>6. 主要研究者GCP培训证书<br>7. 专业组人员名单		
申办者/任务下达单位	无		
临床研究单位	广东省中医院(健康状态辨识与中医治未病创新团队)		
主要研究者	杨志敏		
伦理审查方式	会议审查		
审查委员	韩云,许丽梅,肖静,萧蕙,李晓彦,刘博,朱飞,冯昊禧		
审查意见	根据国家食品药品监督管理局《药物临床试验质量管理规范》《医疗器械临床试验质量管理规范》《药物临床试验伦理审查工作指导原则》，卫计委《涉及人的生物医学研究伦理审查办法》《干细胞临床研究管理办法（试行）》，国家中医药管理局《中医药临床研究伦理审查平台建设规范》，以及世界医学会《赫尔辛基宣言》和国际医学科学组织委员会《涉及人的健康相关研究国际伦理准则》的伦理原则，经本伦理委员会审查，同意按照上述临床研究方案和上述已通过审查的文件进行临床研究。		
伦理委员会声明	本批件将在各中心机构及其伦理委员会备案。如果对方案在本机构的可行性（包括研究者的资格与经验、设备与条件等）有不同意见，请及时与本伦理委员会联系。<br>如项目暂停/提前终止/完成临床研究，或发生严重不良事件以及影响研究风险受益比的非预期不良事件，请及时报告伦理委员会。如临床研究方案、知情同意书的任何修改，主要研究者更换，应及时通知伦理委员会，经审查批准后执行。发现影响受试者参加研究意愿的违反方案情况应及时报告。**请在预计跟踪审查日期前1个月提交研究进度/结题报告。**<br>凡涉及中国人类遗传资源、需要报批的研究项目，须在获得中国人类遗传资源管理办公室批准后才能开始研究。非以产品注册为目的的、干预性临床研究，须在医学研究登记备案信息系统（http:114.255.48.20）注册后方可开展研究。		
批件有效期	自2024年03月01日起<br>至2026年03月01日止	跟踪审查频率<br>预计审查日期	12个月<br>2025年03月01日
联系电话	020-81887233转35943，联系人：李晓彦		

主任/副主任委员签字	
广东省中医院伦理委员会（盖章）	
日期：2024年03月01日	

广东省中医院伦理委员会

Ethics Committee of Guangdong Provincial Hospital of Chinese Medicine

会议签到表

Sign-in Sheet of Full IEC Meeting

日期：2024年03月01日

审议项目：舒和颗粒治疗慢性失眠气血失和、心肾两虚证的单中心、双盲、随机、安慰剂对照临床研究

伦理委员会到会委员签名：

姓名	职务	性别	专业情况	签名
杨荣源	主任委员	男	中医内科学、医院管理	请假
韩云	副主任委员	男	中医内科学、中医重症监护学	韩云
刘奕明	委员	女	临床药理	请假
许丽梅	委员	女	中医内科、中医呼吸学	许丽梅
肖静	委员	女	中医妇科学	肖静
萧蕙	委员	女	护理学	萧蕙
李晓彦	委员	女	中医内科学、临床研究方法学、伦理学	李晓彦
刘博	委员	男	药学研究	刘博
朱飞	委员	男	法律代表（非医药学）	朱飞
冯昊禧	委员	男	社区代表（非医药学）	冯昊禧
