# Supplementary material for: Shuhe granule for insomnia: study protocol for a double-blind, randomized, placebo-controlled trial
Source: Front Pharmacol. 2025 Feb 24;16:1542897. doi: 10.3389/fphar.2025.1542897 (PMC11891157; doi:10.3389/fphar.2025.1542897)

**HPLC fingerprint of Shuhe granule.**

**HPLC fingerprint of Shuhe granule.**

**Materials and methods**

0.12 g of Shuhe granules (batch No.2207326, 2308301, 240401, 2312309) were weighed, dissolved in 10 mL of 30 % methanol with ultrasonic treatment for 10 min (250 W, 40 Hz). Sample was centrifuged (20 ℃, 12000 rpm, 10 min) and filtered through 0.22 µm membrane. Paeoniflorin (batch No.110736-202044, China National Institute of Food and Drug Control), and glycyrrhizin (batch No.111610-201908, China National Institute of Food and Drug Control), was dissolved in 30% methanol. The HPLC analysis of Shuhe granule was carried out on a high-pressure liquid chromatography system (Agilent 1260).

The chromatographic separation was performed using a Luna Su C18 (250×4.60 mm, 5 µm) at 30 ℃, Ultrapure water (A), and acetonitrile (B) was used as the mobile phase for analysis. The flow rate was set at 1.0 mL/min. The elution conditions were applied with a gradient program as follows: 95–80% A for 0–15 min, 80% A for 15–35 min, 80-40% A for 35–55 min, 40-95% A for 55–60 min, 95% A for 60–65 min. 10 μL of each sample was injected into the HPLC system for analysis and the fingerprint chromatograms at three different UV wavelengths (230, 237, and 280 nm) were recorded.

**Results**

The HPLC fingerprint of four batches of Shuhe granules were studied, and the retention time of each chromatographic peak was evaluated by determining the relative retention time with two reference compounds (liquiritin and paeoniflorin) (Figure 1). The similarity evaluation System for Chromatographic Fingerprint of Traditional Chinese Medicine (version 2012.130723) was used for evaluation of the fingerprint similarity among the four batches. The fingerprint of S1 sample was taken as the reference atlas. The similarity of HPLC fingerprint of 4 batches of Shuhe granules was all greater than 0.9 in three UV wavelengths detection (Figure 2).

**
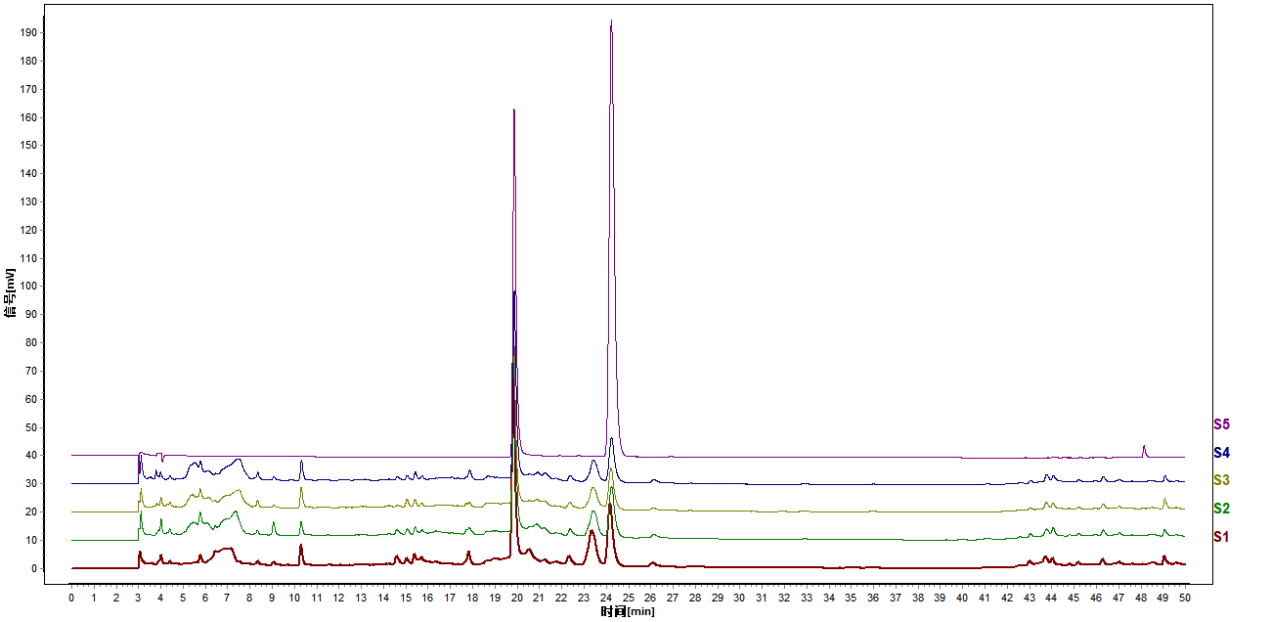
**

**B**

**A**

**Figure 1** HPLC Chromatogram of mixed Reference Solution (S5) and Four Batches of Shuhe Granules (S1-S4). **A:** Paeoniflorin; **B:** liquiritin; **S1:**2201726; **S2:**2308301; **S3:**2404011; and **S4:** 2312309.

**Figure 2** HPLC fingerprint spectra of 4 batches of Shuhe granules at three different wavelengths and the results of fingerprint similarity evaluation. A, 230 nm; B, 237 nm, and C, 280 nm. (**P:** Paeoniflorin; **L:** liquiritin; R: reference sample; **S1**: 2201726; **S2:** 2308301; **S3:** 2404011; and **S4:** 2312309)


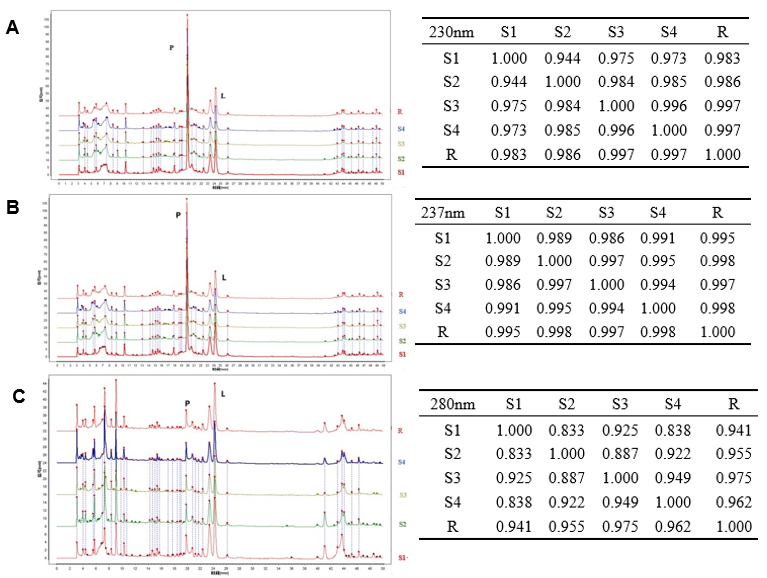

Supplement: Supplementary file 8 [file Supplementaryfile2.docx]
