# Supplementary material for: Shuhe granule for insomnia: study protocol for a double-blind, randomized, placebo-controlled trial
Source: Front Pharmacol. 2025 Feb 24;16:1542897. doi: 10.3389/fphar.2025.1542897 (PMC11891157; doi:10.3389/fphar.2025.1542897)
Supplement: Supplementary file 9 [file Supplementaryfile4.docx]

**Chemical Analysis of Shuhe Granule**

**The chemical investigation of SHUHE by LC-HR MS.**

**(1) Materials and Reagents:** Shuhe granules were manufactured by the Guangzhou Kangyuan Pharmaceutical. Reference substances ferulic acid, glycyrrhizic acid monoammonium salt, ginsenoside Rb1, and ginsenoside Rg1 were from National Institutes for Food and Drug Control. Catechin, adenosine, guanosine, protocatechuic acid, licochalcone A, Vicenin II, uridine, p-hydroxycinnamic acid, p-aminobenzoic acid, 2-methoxybenzoic acid, ginsenoside Re, ginsenoside Rf, coumarin, atractylenolide I, atractylenolide II, atractylenolide III, phenylalanine, and tryptophan were provided by Chengdu PUSH Bio-tech,. Chromatographic grade methanol and acetonitrile were purchased from Merck, Germany. LC-MS grade formic acid was purchased from Thermo-Fisher. The water used in the liquid phase was Millipore purified water (18.2 MΩ).

**(2) Instruments and Experimental Methods:** LC-MS analysis was conducted using a Thermo-Fisher Ultimate 3000 UHPLC system combined with a Q Exactive Orbitrap Plus high-resolution mass spectrometer. The chromatographic column used was a Waters UPLC^TM^ HSS T3 C18 (2.1×100 mm, 1.7 μm) column. The chromatographic conditions were optimized with an elution gradient of acetonitrile (A) - 0.1% formic acid water (B), with the elution program as follows: 0 min 5% A; 8 min 13% A; 12 min 17% A; 25 min 45% A; 30 min 70% A; 35 min 90% A; 40 min 99%. The flow rate was 0.23 mL/min.

Elution was ionized by ESI and entered the Q Exactive Orbitrap high-resolution mass spectrometer in both positive and negative ion detection. The main parameters of the ESI ion source were: spray voltage 3500 V (negative ion -3500 V); capillary temperature 350 ℃; sheath gas: 40; auxiliary gas: 15; other parameters were default. Before the detection, the mass resolution was calibrated to meet the basic requirement of <2 ppm mass accuracy across the full *m/z* range. The mass spectrometer was set to Full scan and DD-MS^2^ scan modes. Full scan ion range between *m/z* 100-1300 with resolution power of 35000. DD-MS^2^ MS/MS scanning settings were: resolution of 17500, precursor ion threshold 1×105, ion selection peak width *m/z* 2.0, the highest peak was selected for secondary scanning with dynamic exclusion for 5 s.

**(3) Chemical composition of Shuhe granule.**

The present study detected more than 120 chemical components of SHUHE in both positive and negative ion modes, covering most of the LC-MS chromatographic peaks (Figure 1-1 and Table 1-1). These chromatographic peaks were mainly identified as: 1. Polar components such as amino acids, oligosaccharides, oligopeptides and their derivatives; 2. Phenolic acids such as ferulic acid, chlorogenic acid, and caffeic acid derivatives; 3. Flavonoids and chromones such as licorice flavonoids; 4. Saponins including ginsenosides and glycyrrhizin; 5. Terpenes and their glycosides represented by paeoniflorin; 6. Other components such as lipids, organic acids, tannins, etc.

Figure 1-1 Shuhe granule LC-MS chromatographic peaks (Top: Negative ion mode; Bottom: Positive ion mode)

Table 1-1 Main Chemical Components of Shuhe Granule

| **No.** | ***t*_R_ (min)** | **precursor ions** | **Elemental composition** | **Mas error**  **ppm** | **Characteristic Fragment ions** | **Identification** |
| --- | --- | --- | --- | --- | --- | --- |
|  | 1.24 | 341.10858 [M-H]^-^  377.08392 [M+Cl]^-^ | C_12_H_21_O_11_  C_12_H_22_O_11_Cl | -1.0 | [341]: 89,101,119,59,71,179,161 | Sucrose |
|  | 1.28 | 136.06178[M+H]^+^ | C_5_H_6_N_5_ | -2.1 | [136]: 94,119 | Adenine |
|  | 1.60 | 136.06178[M+H]^+^ | C_5_H_6_N_5_ | 0.1 | [136]: 94,119 | Hypoxanthine |
|  | 1.69 | 130.04979 [M+H]^+^ | C_5_H_8_O_3_N | 0.5 | [130]: 84 | Pyroglutamic acid |
|  | 1.69 | 191.01906 [M-H]^-^ | C_6_H_7_O_7_ | -3.4 | [191]:111,102,87,129 | Citric acid |
|  | 1.69 | 268.10376 [M+H]^+^ | C_10_H_14_O_4_N_5_ | -1.0 | [268]: 136 | Adenosine |
|  | 1.69 | 389.10870[M-H]^-^ | C_16_H_21_O_11_ | -0.6 | 227,209,191,165,147,135,113 | Monotropein |
|  | 1.83 | 282.08459 [M-H]^-^  565.17450[2M-H]^-^  284.09839 [M+H]^+^ | C_10_H_12_O_5_N_5_  C_20_H_25_O_10_N_10_  C_10_H_14_O_5_N_5_ | 0.7  0.0  -1.9 | [284]: 152 | Guanosine |
|  | 1.86 | 294.15417 [M+H]^+^ | C_12_H_24_O_7_N | -1.9 | [294]: 276,258,230,212,132,86 | Leucyl-glucosyl-carbohydrate |
|  | 2.46 | 243.06114[M-H]^-^ | C_9_H_11_O_6_N_2_ | -0.1 |  | Uridine |
|  | 3.50 | 331.06750 [M-H]^-^ | C_13_H_15_O_10_ | 1.5 | [331]: 169,211,271,125 | Gallic acid glucoside |
|  | 3.86 | 493.12054 [M-H]^-^ | C_19_H_25_O_15_ | 1.7 | [493]: 313,169,271 | Digalloyl glucose |
|  | 4.03 | 166.08591 [M+H]^+^ | C_9_H_12_O_2_N | -2.0 | [166]: 120,131 | Phenylalanine |
|  | 4.09 | 283.13953 [M+H]^+^  281.12567 [M-H]^-^ | C_12_H_19_O_4_N_4_  C_12_H_17_O_4_N_4_ | -1.9  0.5 | [283]: 112,265,248,206,162,134 | Amino acid derivatives |
|  | 4.11 | 331.06750 [M-H]^-^ | C_13_H_15_O_10_ | 1.5 | [331]: 169,211,271,125 | Gallic acid glucoside |
|  | 4.14 | 493.12054 [M-H]^-^ | C_19_H_25_O_15_ | 1.7 | [493]: 313,169,271 | Digalloyl glucose |
|  | 4.50 | 353.14383[M-H]^-^  399.15085[M+HCOO]^-^ | C_14_H_25_O_10_  C_15_H_27_O_12_ | -1.1  0.1 | [353]: 221,161 | Isopropyl- pentosyl-hexoside |
|  | 4.90 | 315.07239 [M-H]^-^ | C_13_H_15_O_9_ | 0.7 | [315]: 153,152, 109,108 | Dihydroxybenzoic acid glucoside |
|  | 4.90 | 315.07242 [M-H]^-^ | C_13_H_15_O_9_ | 1.3 | [315]: 152, 153, 108 | Protocatechuic acid glucoside |
|  | 5.46 | 253.12921 [M+H]^+^ | C_11_H_17_O_3_N_4_ | -1.2 | [253]: 166,165,70,236,122,181, 208,191, 225,112 | L-Histidyl-L-proline |
|  | 6.02 | 153.01840 [M-H]^-^ | C_7_H_5_O_4_ | -6.0 | [153]: 109 | Protocatechuic acid |
|  | 6.05 | 315.07242 [M-H]^-^ | C_13_H_15_O_9_ | 1.3 | [315]: 152, 153, 108 | Protocatechuic acid glucoside |
|  | 6.21 | 407.15601 [M+HCOO]^-^  361.15030 [M-H]^-^ | C_17_H_27_O_11_  C_16_H_25_O_9_ | 1.2  0.9 | [361]: 199,119,89,71,59,181 | 6-O-β-D-glucopyranosyl-lactinoside |
|  | 6.55 | 367.14874 [M+H]^+^ | C_17_H_23_O_7_N_2_ | -3.3 | [367]: 188,229,332, | Tryptophan mannose glycoside |
|  | 6.60 | 515.13940[M-H]^-^ | C_22_H_27_O_14_ | -0.1 | [515]: 353,341,323,191,161 | Chlorogenic acid glucoside |
|  | 6.92 | 353.08786 [M-H]^-^  355.10104 [M+H]^+^ | C_16_H_17_O_9_  C_16_H_19_O_9_ | 0.2  0.1 | [353]: 191,179,135,179  [355]: 163 | Chlorogenic acid |
|  | 7.09 | 205.09691[M+H]^+^  203.08119[M-H]^-^ | C_11_H_13_O_2_N_2_  C_11_H_11_O2N_2_ | -1.2  -1.5 | [205]: 188, 146 | Tryptophan* |
|  | 7.30 | 341.08752 [M-H]^-^ | C_15_H_17_O_9_ | -0.8 | [341]: 179,135 | Caffeic acid-O-glucoside |
|  | 8.35 | 175.05977[M-H]^-^ | C_7_H_11_O_5_ | -1.8 | [175]: 115,85,157,131 | Hydroxyheptanoic acid |
|  | 8.39 | 443.19116[M-H]^-^ | C_21_H_31_O_10_ | 0 | [443]: 59,71,89,101,113,119,161,281 | dihydrophaseic acid-O-glucoside |
|  | 8.48 | 515.13940[M-H]^-^ | C_22_H_27_O_14_ | -0.1 | [515]: 353,341,323,191,161 | Chlorogenic acid glucoside |
|  | 8.66 | 209.04416 [M-H]^-^  165.05417 [M-CO_2_-H]^-^ | C_10_H_9_O_5_  C_9_H_9_O_3_ | -0.2  -0.5 | [209]: 165,121,59,79 | hydroxyferulic acid |
|  | 8.69 | 495.15176 [M-H]^-^ | C_23_H_27_O_12_ | 1.7 | [495]: 137 | Hydroxypaeoniflorin |
|  | 8.89 | 355.10208[M-H]^-^  401.10748[M+HCOO]^-^  195.06508[M-glu+H]^+^ | C_16_H_19_O9  C_17_H21O_11_ | -0.7  -0.9 | [355]: 193,149,134,178 | Ferulic acid glucoside |
|  | 9.01 | 345.15512 [M-H]^-^  391.16098[M+HCOO]^-^  369.15121 [M+Na]^+^ | C_16_H_25_O_8_  C_17_H_27_O_10_  C_16_H_26_O_8_Na | 0.7  1.1  -2.1 | [345]: 183,89,59,71,119,101 | glucopyranosyl-deoxylactinoside |
|  | 9.37 | 341.08752 [M-H]^-^ | C_15_H_17_O_9_ | -0.8 | [341]: 179,135 | Caffeic acid-O-glucoside |
|  | 9.57 | 515.13940[M-H]^-^ | C_22_H_27_O_14_ | -0.1 | [515]: 353,341,323,191,161 | Chlorogenic acid glucoside |
|  | 9.63 | 495.15176 [M-H]^-^ | C_23_H_27_O_12_ | 1.7 | [495]: 137 | Hydroxypaeoniflorin |
|  | 9.82 | 353.08786 [M-H]^-^  355.10104 [M+H]^+^ | C_16_H_17_O_9_  C_16_H_19_O_9_ | 0.2  0.1 | [353]: 191,179,135,179  [355]: 163 | Neochlorogenic acid |
|  | 9.90 | 291.08670 [M+H]^+^ | C_15_H_15_O_6_ | 1.3 | [291]: 139,123,207,165 | Catechin* |
|  | 9.95 | 217.09692[M+H]^+^ | C_12_H_13_O_2_N_2_ | -1.0 | [217]: 144 | lycoperodine-1 |
|  | 10.26 | 367.10205[M-H]^-^  369.11731[M+H]^+^ | C_17_H_19_O_9_  C_17_H_21_O_9_ | -0.8  -1.9 | [367]: 193,137,149,134 | Quinic acid ferulate |
|  | 10.32 | 167.03346[M-H]^-^ | C_8_H_7_O_4_ | -2.5 | [167]: 123 | Vanillic acid |
|  | 10.45 | 353.08786 [M-H]^-^  355.10104 [M+H]^+^ | C_16_H_17_O_9_  C_16_H_19_O_9_ | 0.2  0.1 | [353]: 191,179,135,179  [355]: 163 | Cryptochlorogenic acid |
|  | 10.45 | 417.11786[M-H]^-^  441.11517[M+Na]^+^  257.08099[M+H-glu]^+^ | C_21_H_21_O_9_  C_21_H_22_O_9_Na  C_15_H_13_O_4_ | -0.3  -0.9 | [417]: 255, 135, 153,119 | Liquiritin* |
|  | 11.20 | 179.03357[M-H]^-^ | C_9_H_7_O_4_ | -1.7 | [179]: 135 | Caffeic acid |
|  | 11.26 | 687.21436 [M+HCOO]^-^  641.20844 [M-H]^-^  665.20355 [M+Na]^+^ | C_30_H_39_O_18_  C_29_H_37_O_16_  C_29_H_38_O_16_Na | 1.2  0.8  -2.4 | [641]: 121  [665]: 503,347,543,341 | Paeoniflorin glucoside |
|  | 11.51 | 577.13483[M-H]^-^ | C_30_H_25_O_12_ | -0.4 | [577]: 407,289.07147,125.02283, 245.08139 | Procyanidin B2 |
|  | 12.49 | 687.21436 [M+HCOO]^-^  641.20844 [M-H]^-^  665.20355 [M+Na]^+^ | C_30_H_39_O_18_  C_29_H_37_O_16_  C_29_H_38_O_16_Na | 1.2  0.8  -2.4 | [641]: 121  [665]: 503,347,543,341 | Paeoniflorin glucoside |
|  | 12.49 | 327.10852[M-H]^-^ | C_15_H_19_O_8_ | -0.1 | 165,121,147 | Ophiopogonone A |
|  | 12.63 | 291.08676 [M+H]^+^ | C_15_H_15_O_6_ | 1.5 | [291]: 139,123,207,165 | Epicatechin |
|  | 12.84 | 525.16174 [M+HCOO]^-^  481.16943 [M+H]^+^  503.15128 [M+Na]^+^ | C_24_H_29_O_13_  C_23_H_29_O_11_  C_23_H_29_O_11_Na | 1.4  -2.0  -2.2 | [479]: 121,327 | Albiflorin |
|  | 12.95 | 595.16571[M+H]^+^ | C_27_H_31_O_15_ | -0.1 | [595]: 325,379,409,337,391,403,421,439,295,427 | vicenin II* |
|  | 13.64 | 631.16699 [M-H]^-^ | C_30_H_31_O_15_ | 1.2 | [631]: 169,313,121 | Galloylpaeoniflorin/galloylalbiflorin |
|  | 13.76 | 153.05460[M+H]^+^  135.04408[M-H_2_O+H]^+^ | C_8_H_9_O_3_  C_8_H_7_O_2_ | -0.1  0.2 | [153]: 135 | 2-Methoxybenzoic acid* |
|  | 13.86 | 367.10205[M-H]^-^  369.11731[M+H]^+^ | C_17_H_19_O_9_  C_17_H_21_O_9_ | -0.8  -1.9 | [367]: 191,173,93 | Feruloylquinic acid |
|  | 13.95 | 525.16174 [M+HCOO]^-^  479.15604 [M-H]^-^  503.15128 [M+Na]^+^ | C_24_H_29_O_13_  C_23_H_27_O_11_  C_23_H_29_O_11_Na | 1.3  1.2  -2.0 | [479]: 121,165,327 | Paeoniflorin* |
|  | 13.99 | 179.07019[M+H]^+^ | C_10_H_11_O_3_ | -0.4 | [179]: 147,161,119,133,105 | 4-Hydroxy-2-methoxycinnamaldehyde |
|  | 15.10 | 183.06522[M+H]^+^ | C_9_H_11_O_4_ | 0.2 | [183]: 123,95,155,140 | Syringaldehyde* |
|  | 16.14 | 433.11411 [M-H]^-^ | C_21_H_21_O_10_ | 1.2 | [433]: 271, 151 | Naringin-7-O-β-D-glucoside/naringin-5-O-β-D-glucoside |
|  | 16.17 | 433.11411 [M-H]^-^ | C_21_H_21_O_10_ | 1.2 | [433]: 271, 151 | Naringin-7-O-β-D-glucoside/naringin-5-O-β-D-glucoside |
|  | 16.36 | 195.06508[M+H]^+^ | C_10_H_11_O_4_ | -0.5 | [195]: 177,145,107 | Ferulic acid* |
|  | 16.38 | 549.16034[M-H]^-^  257.08099[agl+H]^+^ | C_26_H_29_O_13_  C_15_H_13_O_4_ | 0.1  -0.6 | [549]: 255,135,153,119,419 | Liquiritin apioside/isoliquiritin apioside/Licuraside |
|  | 17..32 | 631.16699 [M-H]^-^ | C_30_H_31_O_15_ | 1.2 | [631]: 169,313,121,271 | Galloylpaeoniflorin/galloylalbiflorin |
|  | 17.75 | 377.15915[M-H]^-^  379.17471 [M+H]^+^ | C_21_H_27_O_9_  C_20_H_25_O_7_  C_20_H_27_O_7_ | 0.2  -0.9  -1.1 | [377]: 362,209,165,151,135 | angelol C |
| 1. 1 | 17.90 | 375.14343[M-H]^-^  377.15912 [M+H]^+^ | C_21_H_25_O_9_  C_20_H_23_O_7_  C_20_H_25_O_7_ | 0.0  -1.0  -1.0 | [375]: 330,290,165,150 | angelol A |
|  | 18.03 | 939.11145 [M-H]^-^ | C_41_H_31_O_26_ | 1.6 | [939]: 169,617,431,295,447,465 | Dicaffeoylquinic acid |
|  | 18.08 | 515.11841[M-H]^-^ | C_25_H_23_O_12_ | 0 | [515]: 191,353,173,179 | Galloylpaeoniflorin/galloylalbiflorin |
|  | 18.54 | 631.16699 [M-H]^-^ | C_30_H_31_O_15_ | 1.2 | [631]: 169,313,121 | Dicaffeoylquinic acid |
|  | 18.57 | 515.11841[M-H]^-^ | C_25_H_23_O_12_ | 0 | [515]: 191,353,173,179 | 3,5-Dimethoxy-4-hydroxycinnamaldehyde |
|  | 18.61 | 209.08061[M+H]^+^ | C_11_H_13_O_4_ | -1.0 | [209]: 177,191,149,121,135 | Dicaffeoylquinic acid |
|  | 19.18 | 961.53784 [M-H]^-^  1007.54425 [M+HCOO]^-^ | C_48_H_81_O_19_  C_49_H_83_O_21_ | 0.1  1.0 | [1007]: 961,637,475, 799,781,619 | 20-O-Glucoginsenoside Rf/ Ginsenoside Re2 |
|  | 19.21 | 515.11841[M-H]^-^ | C_25_H_23_O_12_ | 0 | [515]: 191,353,173,179 | Dicaffeoylquinic acid |
|  | 19.21 | 509.16711 [M-H]^-^ | C_24_H_29_O_12_ | 1.7 | [509]: 121 | Monoterpenoid glycoside |
|  | 19.54 | 961.53784 [M-H]^-^  1007.54425 [M+HCOO]^-^ | C_48_H_81_O_19_  C_49_H_83_O_21_ | 0.1  1.0 | [1007]: 961,637,475,799, 781,619 | 20-O-Glucoginsenoside Rf/ Ginsenoside Re2 |
|  | 19.55 | 147.04405[M+H]^+^ | C_9_H_7_O_2_ | 0 | [147]: 103,91 | Coumarin* |
|  | 19.86 | 549.16034[M-H]^-^  257.08099[agl+H]^+^ | C_26_H_29_O_13_  C_15_H_13_O_4_ | 0.1  -0.6 | [549]: 255,135,153,119,419 | Liquiritin apioside/isoliquiritin apioside/Licuraside |
|  | 20.06 | 845.49030 [M+HCOO]^-^ | C_43_H_73_O_16_ | 1.0 | [845]: 475,637,757,665 | Ginsenoside Rg1* |
|  | 20.18 | 549.16034[M-H]^-^  257.08099[agl+H]^+^ | C_26_H_29_O_13_  C_15_H_13_O_4_ | 0.1  -0.6 | [549]: 255,135,153,119,419 | Liquiritin apioside/isoliquiritin apioside/Licuraside |
|  | 20.28 | 431.13327 [M+H]^+^  475.12354[M+HCOO]^-^ | C_22_H_23_O_9_  C_23_H_23_O_11_ | -0.9  0.1 | [431]: 269 | Mangiferin |
|  | 20.38 | 417.11792[M-H]^-^  419.13321 [M+H]^+^ | C_21_H_21_O_9_  C_21_H_23_O_9_ | -0.2  -1.1 | [417]: 255, 135, 153,119 | Isoliquiritin |
|  | 20.76 | 417.11792[M-H]^-^  419.13321 [M+H]^+^ | C_21_H_21_O_9_  C_21_H_23_O_9_ | -0.2  -1.1 | [417]: 255, 135, 153,119 | Neo-liquiritin |
|  | 21.03 | 491.21426 [M+HCOO]^-^  445.20825 [M-H]^-^ | C_22_H_35_O_12_  C_21_H_33_O_10_ | 1.9  1.4 | [445]: 89,71,131,149,191,233,293,283 | β-pinen-10-yl-β-vicianoside |
|  | 21.37 | 247.13293[M+H]^+^ | C_15_H_19_O_3_ | 0.3 | [247]: 201,173,111,219 | 8,9-Epoxycostunolide |
|  | 21.99 | 375.18097 [M-H]^-^ | C_21_H_27_O_6_ | 0.7 | [375]: 135,109,179,165,360 | Octahydrocurcumin |
|  | 23.11 | 983.44659[M-H]^-^ | C_48_H_71_O_21_ | -1.7 | [983]: 821,351,645,193 | Glycyrrhizin |
|  | 23.41 | 373.16519 [M-H]^-^ | C_21_H_25_O_6_ | 0.6 | [373]: 179,193,165 | Hexahydrocurcumin |
|  | 23.44 | 629.18805 [M+HCOO]^-^  583.18262 [M-H]^-^ | C_31_H_33_O_14_  C_30_H_31_O_12_ | 1.6  1.6 | [583]: 121 | Benzoylalbiflorin |
|  | 23.51 | 249.14867[M+H]^+^ 231.13789[M+H-H_2_O]^+^ | C_15_H_19_O_2_  C_15_H_21_O_3_ | -0.2  0.5 | [249]: 231,163 | Atractylenolide III* |
|  | 23.56 | 799.48578 M-H]^-^  845.49127 [M+HCOO]^-^ | C_42_H_71_O_14_  C_43_H_73_O_16_ | 0.9  0.9 | [845]: 475,637,799 | Ginsenoside Rf* |
|  | 23.80 | 629.18805 [M+HCOO]^-^  583.18262 [M-H]^-^ | C_31_H_33_O_14_  C_30_H_31_O_12_ | 1.6  1.6 | [583]: 121 | Benzoylpaeoniflorin |
|  | 24.00 | 879.39960[M-H]^-^ | C_44_H_63_O_18_ | -1.5 | [879]: 351,193,113,643 | Glycyrrhizin |
|  | 24.03 | 983.44659[M-H]^-^ | C_48_H_71_O_21_ | -1.7 | [983]: 821,351,645,193 | Glycyrrhizin isomer |
|  | 24.16 | 1077.58508 [M-H]^-^  1123.59094 [M+HCOO]^-^ | C_53_H_89_O_22_  C_54_H_91_O_24_ | 0.0  0.4 | [1123]: 1077,945,783,621,459, 915,765 | Ginsenoside Rb2/Rb3 |
|  | 24.18 | 1107.59595 [M-H]^-^  1153.60156 [M+HCOO]^-^ | C_54_H_91_O_23_  C_55_H_93_O_25_ | 0.3  0.4 | [1153]: 1107, 945,783,621,459 | Ginsenoside Rb1* |
|  | 24.27 | 837.38898[M-H]^-^  839.40485[M+H]^+^ | C_42_H_61_O_17_  C_42_H_63_O_17_ | -1.6  -1.3 | [837]: 351,193,113,661,485 | Glycyrrhizin G2/its isomers |
|  | 24.44 | 1193.59668 [M-H]^-^  1149.60669 [M-CO_2_]^-^ | C_57_H_93_O_26_  C_56_H_93_O_24_ | 0.6  0.5 | [1193]: 1107,945,783,621,459 | Malonylginsenoside Rb1 |
|  | 24.90 | 1163.58594 [M+HCOO]^-^  1119.59595 [M-CO_2_]^-^ | C_56_H_91_O_25_  C_55_H_91_O_23_ | 0.4  0.3 | [1163]: 1077,945,783,621,459, 1059,915,765 | Malonylginsenoside Rb2 |
|  | 24.95 | 979.48682 [M+H]^+^  439.35709 [agl+H-H_2_O]^+^  955.49127 [M-H]^-^ | C_48_H_76_O_19_Na  C_30_H_47_O_2_  C_48_H_75_O_19_ | -0.5  0.5 | [955]: 793,731,613,569,523,775 | Ginsenoside Ro |
|  | 25.05 | 1077.58508 [M-H]^-^  1123.59094 [M+HCOO]^-^ | C_53_H_89_O_22_  C_54_H_91_O_24_ | 0.0  0.4 | [1123]: 1077,945,783,621, 459,915,765 | Ginsenoside Rb2/Rb3 |
|  | 25.31 | 1163.58594 [M+HCOO]^-^  1119.59595 [M-CO_2_]^-^ | C_56_H_91_O_25_  C_55_H_91_O_23_ | 0.4  0.3 | [1163]: 1077,945,783,621,459, 1059,915,765 | Malonylginsenoside Rb3 |
|  | 25.48 | 837.38898[M-H]^-^  839.40485[M+H]^+^ | C_42_H_61_O_17_  C_42_H_63_O_17_ | -1.6  -1.3 | [837]: 351,193,113,661,485 | Glycyrrhizin G2/isomer |
|  | 26.05 | 837.38898[M-H]^-^  839.40485[M+H]^+^ | C_42_H_61_O_17_  C_42_H_63_O_17_ | -1.6  -1.3 | [837]: 351,193,113,661,485 | Glycyrrhizin G2/isomer |
|  | 26.05 | 945.54370 [M-H]^-^  991.54944 [M+HCOO]^-^ | C_48_H_81_O_18_  C_49_H_83_O_20_ | 0.8  1.1 | [945]: 475,637,783,619,765  [991]: 475,637,783,619,765,945 | Ginsenoside Re* |
|  | 26.15 | 163.07520[M+H]^+^ | C_10_H_11_O_2_ | -0.9 | [163]: 55,107,135,145,105 | Methoxycinnamaldehyde |
|  | 26.17 | 985.46240[M-H]^-^ | C_48_H_73_O_21_ | -1.5 | [985]: 497,321,339,435,663,645 | Glycyrrhizin |
|  | 26.25 | 255.06522[M-H]^-^  257.08124[M+H]^+^ | C_15_H_11_O_4_  C_15_H_13_O_4_ | 0.1  1.5 | [255]: 119,135,153 | Liquiritin |
|  | 26.45 | 837.38898[M-H]^-^  839.40485[M+H]^+^ | C_42_H_61_O_17_  C_42_H_63_O_17_ | -1.6  -1.3 | [837]: 351,193,113,661,485 | Glycyrrhizin G2/isomer |
|  | 26.57 | 267.06540[M-H]^-^ | C_16_H_11_O_4_ | 0.8 | [267]: 252 | Mangiferin |
|  | 26.57 | 821.39417[M-H]^-^  823.40936[M+H]^+^ | C_42_H_61_O_16_  C_42_H_63_O_16_ | -1.5  -2.0 | [821]: 351,193,113,175,289  [823]: 453,471,435,407 | Glycyrrhizic acid* |
|  | 26.83 | 985.46240[M-H]^-^ | C_48_H_73_O_21_ | -1.5 | [985]: 497,321,339,435,663,645 | Glycyrrhizin |
|  | 27.70 | 821.39417[M-H]^-^  823.40936[M+H]^+^ | C_42_H_61_O_16_  C_42_H_63_O_16_ | -1.5  -2.0 | [821]: 351,193,113,175,289  [823]: 453,471,435,407 | Glycyrrhizin K2/Glycyrrhizin H2 |
|  | 28.01 | 821.39417[M-H]^-^  823.40936[M+H]^+^ | C_42_H_61_O_16_  C_42_H_63_O_16_ | -1.5  -2.0 | [821]: 351,193,113,175,289  [823]: 453,471,435,407 | Glycyrrhizin K2/Glycyrrhizin H2 |
|  | 28.33 | 823.41022[M-H]^-^ | C_42_H_63_O_16_ | -1.0 | [823]: 351,193,113,175,289 | Larglycyrrhizin C/Glycyrrhizin J2 |
|  | 28.85 | 277.17978 [M+H]^+^ | C_17_H_25_O_3_ | -0.1 | [277]: 177,137,146 | [6]-Gingerol* |
|  | 28.87 | 823.41022[M-H]^-^ | C_42_H_63_O_16_ | -1.0 | [823]: 351,193,113,175,289 | Larglycyrrhizin C/Glycyrrhizin J2 |
|  | 29.52 | 367.11722[M-H]^-^ | C_21_H_19_O_6_ | -1.0 | [367]: 309,297,339,284 | Glycycoumarin |
|  | 30.37 | 313.23755[M-H]^-^ | C_18_H_33_O_4_ | 0.7 | [313]: 201,295,77,171 | Octadecanedioic acid |
|  | 30.63 | 353.10168[M-H]^-^ | C_20_H_17_O_6_ | -0.8 | [353]: 125,227 | Isolicoflavonol |
|  | 31.01 | 239.10683[M+H]^+^ | C_16_H_15_O_2_ | 0.7 | [239]: 161,221,105,133,193 | 1,4-Diphenylbutadione |
|  | 31.08 | 313.23755[M-H]^-^ | C_18_H_33_O_4_ | 0.7 | [313]: 201,295,77,171 | Octadecanedioic acid |
|  | 32.08 | 351.08621[M-H]^-^ | C_20_H_15_O_6_ | -0.3 | [351]: 283 | gancaonin M |
|  | 32.25 | 293.21143[M-H]^-^ | C_18_H_29_O_3_ | 1.0 | [293]: 275,235,171,121 | Hydroxylinolenic acid |
|  | 33.35 | 295.22705[M-H]^-^  279.23242 [M+H-H2O]^+^ | C_18_H_31_O_3_  C_18_H_31_O_2_ | 0.9  2.0 | [295]: 277,195,171 | Hydroxylinoleic acid |
|  | 33.97 | 293.21143[M-H]^-^ | C_18_H_29_O_3_ | 1.0 | [293]: 275,235,171,121 | Hydroxylinolenic acid |

*Compared with reference compounds
